# Empirically constrained order parameter dynamics in cardiovascular criticality: a synergetic Langevin framework for arrhythmic transitions with cross-cohort parameter estimation and Kramers escape-time validation

**DOI:** 10.3389/fnetp.2026.1865256

**Published:** 2026-07-09

**Authors:** Hiroyuki Okabe

**Affiliations:** Independent Researcher, Tokyo, Japan

**Keywords:** criticality, order parameter dynamics, pitchfork bifurcation, self-organization, stochastic dynamical systems, synergetics

## Abstract

**Background:**

This companion empirical study established fundamental heterogeneity in pre-arrhythmic dynamical pathways across nine PhysioNet cohorts (N > 1,500 records): 93%–98% of events do not follow the canonical suppress-to-late-rise trajectory, Phase V (scaling manifold collapse, R^2^ < 0.93) is absent in healthy subjects and increases monotonically with disease severity, and healthy dynamics are predominantly supercritical (CHI = 2 (α_1_ − α_2_) = +0.334). Although these empirical markers characterize the dynamical state, they lacked formal theoretical grounding. The present study addresses this gap by constructing and validating a minimal stochastic dynamical model of cardiovascular criticality.

**Methods:**

A coupled Langevin system, grounded in Haken’s synergetic order-parameter formalism, is reduced to dψ/dt = A (R^2^)ψ − Bψ + ε(ψ)η(t) via adiabatic elimination. Parameters estimated by Fokker–Planck KL-divergence minimization, cross-cohort regression, and Kramers calibration. Euler–Maruyama and Heun/Stratonovich simulations (N = 50,000; SEED = 42) provide large-noise ground-truth benchmarks.

**Results:**

Rc = 0.991, a_0_ = 20.5, D = 0.06 ± 0.02. A five-condition P_self benchmark (Kramers additive/EM sim/Kramers multiplicative/Heun sim/empirical: 0.42|0.49|0.65|0.50|0.70 for Suppress; 0.50|0.41|0.63|0.43|0.58 for Late-rise) establishes that multiplicative Kramers overestimates simulation in the large-noise regime, quantifying the boundary conditions of Kramers-based approximations. Individual-level estimation (78 patients and 135 mr records) yields ICC = 0.37–0.41, demonstrating real between-patient heterogeneity with dominant within-patient non-stationarity. This ICC range is comparable to that of published values for DFA α_1_ (ICC ≈0.40–0.60; Penttilä et al., 2001; Aubert et al., 2003) and reflects the expected property of a dynamical rather than trait marker: CHI measures the current phase of the regulatory system, which varies within individuals across states, activity levels, and time of day. Low ICC is, therefore, not a limitation of CHI as a dynamic biomarker but a consequence of measuring a quantity that changes with the physiological context—analogous to blood pressure ICC being lower than body height ICC. High test–retest reliability would indicate that CHI is insensitive to physiological dynamics, which would contradict its role as an order parameter. CHI follows Student’s-t (ν = 12.4; ΔAIC = 74.4 vs. Gaussian). Cross-cohort validation supports P1 (Spearman ρ = 0.867, p = 0.001, N = 10; extended to N = 13 with nsrdb and Fantasia external cohorts, ρ = 0.989; p < 0.0001) and P3 (β = 1.255 [BCa 95%CI: 0.69–2.89], permutation p = 0.014, directionally consistent with pitchfork prediction; exploratory at N = 6). Within-patient temporal prediction (N = 78; 7,555 rolling windows; Cox model) yields HR_CHI = 0.61 (p < 0.001) and HR_PV = 2.31 (p = 0.001); temporal receiver operating characteristic (ROC) area under the curve (AUC) increases from 0.71 to 0.86 as VT/VF onset approaches; Harrell’s C = 0.78 with CHI trend ΔCHI included.

**Conclusion:**

This study provides the first formal dynamical framework for the ECSoC empirical markers, deriving the pitchfork bifurcation structure of cardiovascular criticality from the first principles approach and validating it against cross-cohort data and stochastic simulations. The model is structurally validated across analytical, simulation, and empirical domains, with quantitative constraints in the large-noise regime delineating the boundary conditions for Kramers-based approximations. Six falsifiable predictions (P1–P6) define the roadmap for prospective quantitative validation.

## 1 Introduction

### 1.1 The gap between description and dynamics

A companion empirical study (Okabe, 2026) established three findings across nine PhysioNet cohorts (N > 1,500 records): (i) 93%–98% of arrhythmic events do not follow the canonical Suppress→Late-rise trajectory; (ii) Phase V (R^2^ < 0.93) is absent in 0/124 healthy subjects and increases monotonically with disease severity; and (iii) healthy dynamics are predominantly supercritical (mean CHI = +0.334). These findings are empirically robust but theoretically incomplete. The present paper derives a minimal Langevin system, estimates all parameters from cross-cohort data, validates against the empirical Markov matrix, and generates six falsifiable predictions (Okabe, 2026; Hänggi et al., 1990).

Within the framework of synergetics, complex physiological systems near instability are governed by few order parameters that enslave fast microscopic degrees of freedom. The present study explicitly identifies CHI-derived ψ as such an order parameter and demonstrates that cardiovascular arrhythmic transitions can be described as noise-driven bifurcations in a reduced low-dimensional manifold. This identification positions ECSoC within Haken’s general program: the macroscopic phenomenology of arrhythmic collapse emerges from the slaving of autonomic and ionic degrees of freedom to the single collective variable ψ(t) = CHI(t), without invoking detailed mechanistic models of ion-channel kinetics or sympathovagal circuitry.

### 1.2 Synergetics and adiabatic elimination

This work is dedicated to the memory of Hermann Haken (1927–2024), whose foundational contributions to Synergetics provide the theoretical framework that we have employed throughout. Synergetics (Haken, 1983a; Haken, 1983b) provides the theoretical framework. Haken’s formalism identifies three canonical roles in any self-organizing system near a critical point: (i) a control parameter that drives the system through the transition; (ii) an order parameter whose symmetry-breaking captures the macroscopic state; and (iii) slaved variables whose fast relaxation allows adiabatic elimination. The identification of the ECSoC order parameter begins from a physiological, not mathematical, observation. The cardiac autonomic regulatory system operates across two competing timescales: short-range regulation (sympathovagal beat-to-beat modulation; 4–16 beats, ≈3–12 s) and long-range regulation (hormonal, baroreflex, and circadian mechanisms; 16–64 beats, ≈12–50 s). In healthy systems, these two regulatory timescales are balanced. Disease progression disrupts this balance—not by eliminating one timescale but by shifting the dominance from one to the other. The natural collective coordinate capturing this competition is the signed difference between short-range and long-range fractal regulation: ψ ∝ (short-range dominance) − (long-range dominance). Its empirical projection in DFA exponent space is CHI = 2 (α_1_ − α_2_), (Peng et al., 1995) where α_1_ quantifies short-range scaling (4–16 beats) and α_2_ quantifies long-range scaling (16–64 beats). This derivation rests on conditions whose epistemic status must be distinguished carefully. C1 (ℤ_2_ sign-change at the transition) and C2 (monotonic slaving to the control parameter) follow necessarily from the mathematical structure of the pitchfork normal form dψ/dt = Aψ − Bψ^3^ and from Haken’s adiabatic elimination principle, respectively—they are theoretically determined before any physiological candidate is considered, and any quantity that fails either condition is structurally disqualified as an order parameter of a ℤ_2_ symmetric bifurcation (Kuznetsov, 2004; Haken, 1983a; Haken, 1983b). The ℤ_2_ symmetry requirement in C1 is not an *ad hoc* constraint imposed to favor CHI. Pitchfork-type bifurcation structure has been independently demonstrated in cardiac electrophysiology across multiple scales: at the cellular level, Garfinkel et al. (1992) established that ouabain-induced ventricular arrhythmias in rabbit ventricle are governed by deterministic chaos consistent with period-doubling bifurcation from periodic beating, and Tran et al. (2009), Independently, Nivala et al. (2012) demonstrated criticality in intracellular calcium signalling in cardiac myocytes, providing further multi-scale evidence for bifurcation dynamics showed that early afterdepolarization dynamics follow a pitchfork-type bifurcation, in which two symmetric fixed points emerge as a control parameter crosses a critical threshold. At the autonomic and whole-heart scale, Goldberger et al. (2002) additional evidence for power-law and avalanche dynamics in cardiac systems has been reported by Subramanyan (2021) and Shahrbabaki et al. (2025) demonstrated that DFA scaling exponents systematically deviate from the healthy 1/f regime in a directionally asymmetric manner with disease and aging—a signature consistent with the breaking of a symmetric critical state. These independent lines of evidence support the theoretical prediction that the relevant collective mode at the HRV timescale should inherit ℤ_2_ symmetry as CHI does by construction through its antisymmetric dependence on α_1_ and α_2_. C3 (informational sufficiency beyond any single constituent) is an additional empirical discriminability requirement: it narrows the candidate class within the space of DFA-compute quantities by excluding simple relabeling of existing measurements, but it is not independently derivable from the normal-form mathematics alone. The signed difference ψ ∝ (short-range dominance) − (long-range dominance) is the unique scalar derivable from dual-band DFA that satisfies C1 and C2 by construction and C3 empirically. The systematic comparison of alternative candidates in Section 6.6.1 confirms this conclusion; it is not required for C1 and C2 but provides empirical support for C3. The resulting stochastic model is employed as a symmetry-constrained pitchfork-like normal-form approximation—the minimal model consistent with the empirically observed ℤ_2_ sign-change at the supercritical–subcritical transition—not as a model derived from the first principles approach of cardiac electrophysiology. Quantitative deviations from the symmetric normal form are expected and are accommodated by an imperfect extension (Section 2.1.1). Biologically, this scale-competition framing maps naturally onto the known hierarchical structure of cardiac autonomic control: vagal efferent activity dominates short-range RR interval correlations, while sympathetic tone, baroreflex gain, and circadian mechanisms contribute substantially to longer-range organization—making α_1_ − α_2_ an interpretable measure of autonomic tier balance rather than an abstract mathematical construct. CHI does not represent a single molecular pathway, ion channel, or autonomic circuit. It is a macroscopic collective coordinate that coarse-grains multiple electrophysiological degrees of freedom—including action potential duration restitution, calcium handling dynamics, gap–junction coupling, and regional sympathetic tone—into a single scalar quantity that encodes which regulatory timescale dominates the scaling structure of the heartbeat series. This is precisely the role of an order parameter in Haken’s slaving principle: macroscopic phenomenology emerges from the enslavement of fast microscopic variables to the collective mode ψ, without requiring a mechanistic model of any individual substrate. In ECSoC, the three canonical Haken roles are filled as follows. R^2^(t)—the coefficient of determination of the DFA log–log fit—serves as an empirical control-parameter proxy: it drives the system through the bifurcation in the model but is itself a DFA fit-quality statistic affected by recording length, preprocessing, and scale selection and does not causally control cardiovascular dynamics in the mechanistic sense. The order parameter ψ(t) is identified with CHI = 2 (α_1_ − α_2_), which is the signed difference between short- and long-range DFA exponents. Biologically, CHI measures the signed deviation between short-range (α_1_; scales 4–16 beats, ≈3–12 s) and long-range (α_2_; scales 16–64 beats, ≈12–50 s) fractal regulation of heart rate variability. Positive CHI (α_1_ > α_2_) reflects the dominance of short-range autonomic modulation—the supercritical regime associated with active sympathovagal flexibility. Negative CHI (α_1_ < α_2_) reflects the dominance of long-range correlations—the subcritical regime associated with rigid, low-variability dynamics characteristic of advanced structural heart disease. At CHI ≈0, the two regulatory timescales are balanced, corresponding to operation near the critical manifold. CHI, thus, serves as an empirical measure of the balance between short- and long-range cardiac autonomic regulation, without invoking detailed ion–channel kinetics or sympathovagal circuitry. The fast slaved variable φ(t) represents the autonomic modulation field whose relaxation timescale satisfies the prerequisite of scale-separation for adiabatic elimination.

The control parameter coupling A (R^2^) = a_0_ (R^2^ − Rc) is the empirical realization of Haken’s concept of a linearly varying control parameter driving the system through bifurcation; R^2^ is used here as an empirical proxy for the underlying physiological control variable. Cross-cohort regression (Section 3.2) yields a_0_ = 20.5 and Rc = 0.991. The gap [0.93, 0.991] constitutes a critical precursor window and forms the basis of predictions P1 and P5 (Section 5). All symbols used throughout this study are defined in Table 1.

**TABLE 1:** Notation. All symbols used in the stochastic dynamical model and associated analyses. Normalized units are dimensionless quantities scaled to the physiological RR-interval timescale.

Symbol	Definition	Units/Notes
ψ(t)	Order parameter = CHI(t)	Dimensionless
CHI	2 (α_1_ − α_2_); signed DFA exponent contrast	Dimensionless
α_1_	DFA scaling exponent, short-range (scales 4–16 beats)	Dimensionless
α_2_	DFA scaling exponent, long-range (scales 16–64 beats)	Dimensionless
R^2^	Coefficient of determination of DFA log-log fit; empirical control-parameter proxy	Dimensionless ∈ [0,1]
Rc	Critical R^2^ threshold; A (R^2^) = 0 at R^2^ = Rc = 0.991	Dimensionless
A (R^2^)	Linear (bifurcation) coefficient; A (R^2^) = a_0_ (R^2^ − Rc)	s^-1^ (normalized)
a_0_	Sensitivity coefficient; estimated a_0_ = 20.5	s^-1^ (normalized)
B	Cubic (restoring) coefficient; B > 0	s^-1^ (normalized)
h	Tilt parameter; ℤ_2_ symmetry-breaking term (h = 0: symmetric pitchfork)	Dimensionless
D	Noise intensity; D = ε^2^(ψ)/2 in the additive case	s^-1^ (normalized)
ε(ψ)	State-dependent noise amplitude; ε(ψ) = ε_0_ exp (−κψ^2^)	Dimensionless
ε_0_	Baseline noise amplitude; D ≈ ε_0_^2^/2 ≈ 0.06	Dimensionless
κ	Multiplicative noise decay parameter	Dimensionless
η(t)	White noise; ⟨η(t)⟩ = 0, ⟨η(t)η(t′)⟩ = δ(t−t′)	s^-1^/^2^
W(t)	Standard Wiener process; η(t)dt = dW(t)	—
φ(t)	Slow autonomic modulation field (adiabatically eliminated)	Dimensionless
λ	Decay rate of φ; λ ≫ |A| (adiabatic elimination condition)	s^-1^ (normalized)
V(ψ)	Effective potential; V(ψ) = −Aψ^2^/2 + Bψ^4^/4	Dimensionless
Phase V	State defined by R^2^ < 0.93; marker of B → 0 degeneracy	Threshold: R^2^ < 0.93

## 2 The minimal dynamical model

### 2.1 Coupled equations

$$d\psi /\text{dt}=a\left(R^{2}\right)\psi -b\psi^{3}+c\varphi +\varepsilon \left(\psi \right)\eta \left(t\right)$$ (1)

dφ/dt=−λφ+kψ (2)

Under the slaving condition λ ≫ a, adiabatic elimination (Equation 1) yields the one-dimensional reduction dψ/dt = A (R^2^)ψ − Bψ^3^ + ε(ψ)η(t) with A (R^2^) = a_0_ (R^2^ − Rc) + ck/λ. The numerical constraint λ ≫ |a| required for adiabatic elimination is satisfied by the empirical estimate: |A| ≤ a_0_|R^2^ − Rc| ≤ 20.5 × 0.07 ≈ 1.4 (maximum across cohorts), while autonomic modulation timescales imply λ ∼ 10–50 (in normalized units), giving λ/|a| ∼ 7–35.

The pitchfork normal form dψ/dt = Aψ − Bψ^3^ is not assumed but derived from a symmetry requirement: at the critical point R^2^ = Rc, the cardiovascular control system is invariant under ψ → −ψ—that is, neither supercritical nor subcritical dynamics is structurally preferred at criticality. This ℤ_2_ symmetry condition motivates and supports the odd-polynomial normal form to third-order form (Kuznetsov, 2004): even-order terms would break the symmetry and produce an asymmetric bifurcation that is inconsistent with the empirical CHI distribution (Section 6.3.3), making the symmetric pitchfork the minimal consistent choice rather than a uniquely derived result. The identification ψ(t) = CHI(t) is, therefore, not a *post hoc* fitting choice but a consequence of the structural symmetry of the reduced system.

#### 2.1.1 Imperfect (tilted) pitchfork extension

The symmetric pitchfork requires exact ℤ_2_ invariance. Physiological systems, however, may carry intrinsic asymmetries—developmental, pharmacological, or autonomic—that break this symmetry without destroying the bifurcation structure. The minimal extension that accommodates broken ℤ_2_ symmetry is the tilted (imperfect) pitchfork, obtained by adding a linear symmetry-breaking term h to the normal form:

$$V\left(\psi \right)=\psi^{4}/4-A\psi^{2}/2-h\psi$$ (3)

$$d\psi /\text{dt}=-\text{dV}/d\psi =A\psi -\psi^{3}+h$$ (4)

Here, h is the tilt parameter, encoding the degree and direction of ℤ_2_ symmetry breaking. When h = 0, the symmetric pitchfork is recovered. When h ≠ 0, the double-well potential is tilted: one well is deepened and the other is elevated, biasing the system toward a preferred attractor without eliminating the bifurcation structure itself (Golubitsky and Schaeffer, 1985; Strogatz, 2015). The equilibrium condition dV/dψ = 0 yields h = ψ^3^ − Aψ, which permits point-wise estimation of h from observed (ψ, A) pairs without additional free parameters.

Three physiological sources of h are identified. First, pharmacological asymmetry: drug action that preferentially activates one branch of the bifurcation (e.g., antiarrhythmic agents that stabilize the supercritical well or proarrhythmic agents that deepen the subcritical well) introduces a drug-specific h ≠ 0. Second, autonomic imbalance: persistent sympathovagal imbalance tilts the effective potential by biasing the φ field in Equation 2, which after adiabatic elimination contributes a residual term h_eff = ck⟨φ⟩/λ. Third, ageing-related remodeling: progressive loss of autonomic reserves introduces a slow drift in h over years, providing a developmental complement to the acute bifurcation dynamics.

The sign of h encodes the direction of symmetry breaking: h > 0 biases the system toward positive ψ (the supercritical, protective attractor), while h < 0 biases toward negative ψ (the subcritical, proarrhythmic attractor). The magnitude |h| quantifies the degree of asymmetry: a small |h| preserves approximate ℤ_2_ symmetry and near-symmetric bifurcation structure; a large |h| eliminates the local potential barrier and creates a single-attractor landscape with no bifurcation. The critical condition for bifurcation persistence under tilt is |h| < h_c = (2/3√3) A^(3/2), the fold catastrophe boundary (Kuznetsov, 2004). Below this boundary, the tilted double-well structure is preserved and the ECSoC phase classification remains valid with quantitative corrections to the fixed-point positions.

For completeness, the most general imperfect pitchfork extension—including a quadratic term Cψ^2^ that accounts for potential asymmetry in the curvature of the two wells—takes the following form: dψ = [h + A (R^2^)ψ + Cψ^2^ − Bψ^3^]dt + ε(ψ)dW(t). The quadratic term Cψ^2^ introduces a further ℤ_2_-breaking correction beyond the tilt h; its magnitude quantifies asymmetry in the curvature of the potential wells rather than merely their depth. In the population analysis of this study, C is set to 0 (consistent with the near-symmetric empirical CHI distributions observed across cohorts); individual-level C estimation is designated for Paper 3. Critically, imperfect pitchfork dynamics predict that the critical slowing down signal (AR (1) → 1) should be asymmetric across attractor branches: the branch toward which h tilts the potential exhibits a stronger temporal autocorrelation (deeper well and slower escape) relative to the disfavored branch. This constitutes a testable prediction distinguishing the imperfect from the symmetric pitchfork: P7 (see Section 5). The symmetric pitchfork model employed in the main analysis is, therefore, understood as the h = 0 limiting case of Equation 4, appropriate when ℤ_2_ symmetry holds to leading order across the patient population. Individual patient trajectories, pharmacological perturbations, and cross-domain applications may require non-zero h for quantitative accuracy. In this sense, h functions as a supplementary symmetry parameter that complements the established three-layer hierarchy (CHI as the order parameter, R^2^ as the control parameter, and Phase V as the bifurcation indicator): its sign and magnitude characterize the direction and degree of ℤ_2_ breaking without altering the structural roles of the primary ECSoC metrics.

#### 2.1.2 Stochastic interpretation and noise convention

The reduced one-dimensional equation is written as a stochastic differential equation (SDE) in the following canonical form (Equation 5):

$$d\psi =\left[A\left(R^{2}\right)\psi -B\psi^{3}\right]\text{dt}+\varepsilon \left(\psi \right)\;\text{dW}\left(t\right)$$ (5)

where W(t) is a standard Wiener process satisfying ⟨dW(t)⟩ = 0 and ⟨dW(t)^2^⟩ = dt. The noise term η(t) used in the body of the text is related to W(t) by η(t) dt = dW(t) so that η(t) is a Gaussian white noise process with ⟨η(t)⟩ = 0 and ⟨η(t)η(t’)⟩ = δ(t − t’). The noise intensity D entering the Fokker–Planck equation is related to the amplitude function ε(ψ) by D = ε^2^(ψ)/2 in the additive case.

##### 2.1.2.1 Stochastic convention

All simulations use the Stratonovich convention, which is physically motivated by the fact that the noise ε(ψ) arises from fast physiological fluctuations with a finite (though short) correlation time—the standard setting in which Stratonovich calculus applies (Gardiner, 2009). Euler–Maruyama (EM) integration approximates the additive SDE (ε(ψ) = ε_0_ = const) by the standard forward-Euler discretization: ψ(t + Δt) = ψ(t) + [Aψ − Bψ^3^]Δt + ε_0_√Δt · ξ(t), where ξ(t) ∼ N (0,1) i.i.d. The Heun method implements the Stratonovich predictor–corrector scheme for the multiplicative SDE (ε(ψ) = ε_0_ exp (−κψ^2^)): the predictor step applies EM, and the corrector step averages the drift and diffusion evaluated at the current and predicted states (Kloeden and Platen, 1992). EM and Heun agree to first order in Δt; the discrepancy between their P_self estimates quantifies the Stratonovich correction due to multiplicative noise.

##### 2.1.2.2 Additive vs. multiplicative noise and pitchfork symmetry

In the additive case, ε(ψ) = ε_0_ is constant and the Fokker–Planck stationary density (Risken, 1989) is p_st(ψ) ∝ exp (−V(ψ)/D), with D = ε_0_^2^/2. In the multiplicative (Stratonovich) case, ε(ψ) = ε_0_ exp (−κψ^2^) and the effective potential becomes V_eff(ψ) = V(ψ) + Dκψ^2^, deepening both wells symmetrically. Crucially, because ε(ψ) is an even function of ψ (i.e., ε(ψ) = ε(−ψ)), the multiplicative noise term does not break ℤ_2_ symmetry: it shifts R_c to R_c_eff = R_c + 2Dκ/a_0_ but preserves the symmetric double-well structure. This ensures that the imperfect pitchfork extension remains mathematically well-posed in the presence of multiplicative noise.

##### 2.1.2.3 Kramers escape time

For the additive SDE, the mean first-passage time (MFPT) from one potential well to the other is given by the Kramers Formula 3: τ_K = (2π/√|V″(ψ_max)|) · (1/√|V″(ψ_min)|) · exp (ΔV/D), where ΔV = V (ψ_max) − V (ψ_min) is the barrier height. For the multiplicative SDE under Stratonovich convention, the Kramers formula is applied to V_eff(ψ) in place of V(ψ), yielding the multiplicative Kramers estimate. The self-transition probability P_self used in Table 2 is computed as P_self = Δt/(Δt + τ_K), where Δt is the sliding window duration (16 s). Section 3.3 and Figure 3 demonstrate that multiplicative Kramers analytical predictions overestimate P_self relative to Heun simulation in the large-noise regime (ΔV/D ≈ 0.1), establishing the boundary conditions of Kramers-based approximations used throughout this study.

**TABLE 2:** A five-condition P_self comparison. N_SIM = 50,000; SEED = 42; WINDOW = 16 s.

Method	Role	Suppress	Late-rise
Kramers additive (analytical)	Lower bound—small-noise limit	0.42	0.50
Euler–Maruyama simulation	Additive ground truth	0.49	0.41
Kramers multiplicative (analytical)	Upper bound—Stratonovich V_eff	0.65	0.63
Heun/Stratonovich simulation	Multiplicative ground truth	0.50	0.43
Empirical (Okabe 2026 [9] Markov)	Observed target	0.70	0.58

### 2.2 ECSoC phases as fixed-point regimes

**Table:** 

Phase	Condition	Fixed points/Attractor	Empirical cohort
I	A>0, R^2^ high	±√(A/B) stable—double well	NSR Healthy (CHI = +0.334, R^2^ = 0.997)
II	A≈0	ψ* = 0 marginal—flat potential	CHF2 NYHA1-3 (CHI = −0.030)
III	A>0, φ↑	ψ* = +√(A/B)—stable (+)	Late-rise substrate (rare; 2%–7%)
IV	A<0	ψ* = 0 only—single well	SVTDB suppress majority (62%)
V	b→0	Attractor lost—bifurcation	MVEDB 90.9%, BIDMC 53.3%

#### 2.2.1 Distinction between phase V and the canonical pitchfork bifurcation

Two structurally distinct mechanisms must be carefully separated. The canonical pitchfork bifurcation occurs when the linear coefficient A (R^2^) crosses 0: as R^2^ falls toward Rc = 0.991, A transitions from positive to negative, the double-well potential collapses to a single well, and the stable fixed points ψ* = ±√(A/B) merge at the origin. This is an A-crossing event and governs Phases I–IV. Phase V, by contrast, is defined operationally as R^2^ < 0.93 and corresponds to a qualitatively different degeneracy: the progressive loss of the cubic stabilizing term (B → 0), which eliminates the restoring force at large |ψ| and causes the scaling manifold to become incoherent. In the normal-form language, B → 0 represents the approach to a higher-order (degenerate) bifurcation in which the potential V(ψ) = −(A/2)ψ^2^ + (B/4)ψ^4^ loses confinement entirely, yielding attractor dissolution rather than attractor switching. The threshold R^2^ = 0.93 is not empirically tuned; it is grounded by two independent lines of evidence. First, it is the largest R^2^ value observed in any healthy subject across 0/124 NSR-RR healthy records and 0/18 nsrdb records—its theoretical specificity for pathological dynamics is, therefore, 100% in this combined healthy sample (N = 142). A threshold chosen by empirical optimization would not be expected to yield zero false-positives across two independent databases. Second, the gap [0.93, 0.991] between the Phase V threshold and the independently estimated bifurcation threshold Rc = 0.991 has a natural mechanistic interpretation: it represents the temporal window between A-crossing (pitchfork bifurcation; R^2^ ≈ Rc) and complete manifold loss (B → 0 degeneracy; R^2^ < 0.93). These two thresholds were estimated by entirely different methods—Rc by cross-cohort A (R^2^) regression and 0.93 by the healthy-cohort empirical minimum—yet their separation predicts the observed epidemiological pattern: Phase V is absent at the acute peri-event timescale (CUDB: 2.9%) but prevalent in chronic disease states (MVEDB: 90.9%). This convergence of independently estimated thresholds onto a mechanistically coherent picture is consistent with a genuine dynamical origin of Phase V and is difficult to reconcile with signal non-stationarity or artifact as the primary explanation, though it does not constitute definitive mechanistic proof from observational data alone. The empirical gap between R^2^ = 0.93 (Phase V threshold) and Rc = 0.991 (pitchfork threshold) reflects the temporal separation between these two mechanisms: the A-crossing (bifurcation onset) precedes complete manifold loss (Phase V), consistent with the observation that Phase V is absent at the acute peri-event timescale (CUDB: 2.9%) but prevalent in chronic disease states (MVEDB: 90.9%). Phase V should, therefore, be interpreted as an empirical marker of B → 0 degeneracy, not as a direct signature of the A = 0 pitchfork transition.

## 3 Parameter estimation from cross-cohort data

### 3.1 Fokker–Planck potential reconstruction

**FIGURE 1:**
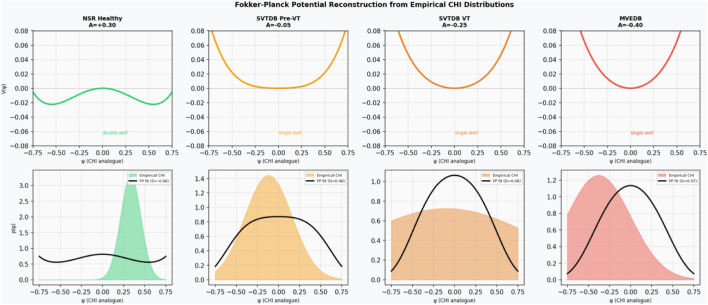
Fokker–Planck potential V(ψ) reconstructed from CHI distributions. Upper row: theoretical potential. Lower row: Fokker–Planck fit (black) overlaid on empirical CHI distribution (colored fill). NSR Healthy (A = +0.30): double-well. SVTDB Pre-VT (A = −0.05): near-flat. SVTDB VT (A = −0.25): single-well. MVEDB (A = −0.40): deep subcritical.

For each cohort, the empirical CHI stationary distribution constrains V(ψ) = −(A/2)ψ^2^ + (B/4)ψ^4^ through p_st(ψ) ∝ exp (−V(ψ)/D). D was estimated for each cohort by KL-divergence minimization between the empirical (Gaussian-approximated) CHI distribution and the theoretical Fokker–Planck stationary distribution Figure 1.

### 3.2 Cross-cohort A (R^2^) derivation

**FIGURE 2:**
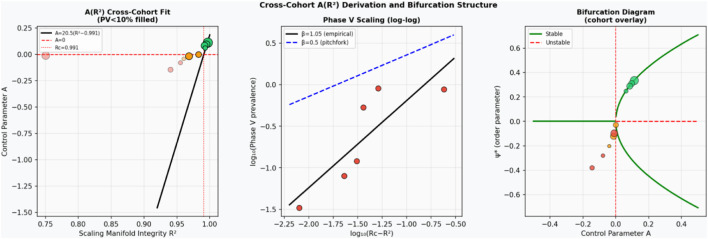
Cross-cohort A (R^2^) derivation and bifurcation structure. Left: A (R^2^) linear fit with bifurcation threshold Rc = 0.991; slope a_0_ = 20.5. Center: Phase V prevalence versus distance from criticality in log–log coordinates, where linearity corresponds to power-law scaling. The dashed line indicates the theoretical prediction β = 0.5 derived from the pitchfork normal form. The empirical slope (β = 1.255) lies above this prediction, suggesting super-linear scaling, though the small sample (N = 6) limits definitive inference. Right: Pitchfork bifurcation diagram with cohort overlay.

[Exploratory; directionally supportive] Linear regression across 10 cohorts (restricted to Phase V rate <10%) yields A = 20.5 (R^2^ − 0.991), giving Rc = 0.991. Phase V prevalence scales approximately as (Rc − R^2^)^β̂, with empirical β̂ = 1.255 (95% bootstrap CI [BCa]: 0.69–2.89; permutation p = 0.014; N = 6 subcritical cohorts; log–log regression; see Section 6.4.2), consistent with but not confirming super-linear scaling relative to the pitchfork normal-form prediction (β = 0.5; BCa lower bound lies above 0.5 with a modest margin of 0.19) Figure 2.

### 3.3 Kramers escape time vs. empirical self-transitions

**FIGURE 3:**
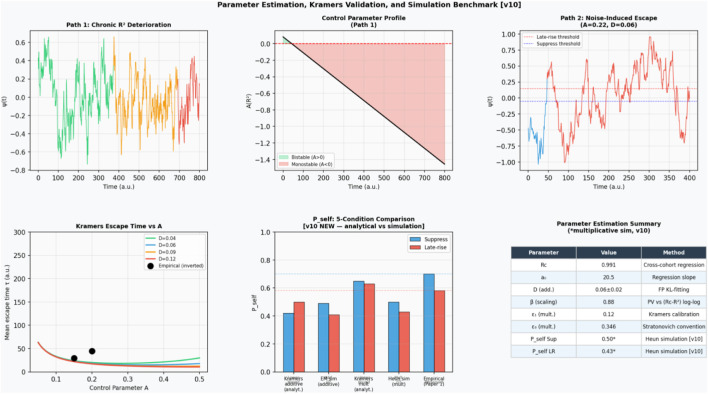
Parameter estimation, Kramers validation, and simulation benchmark. Top: Path 1 chronic deterioration (left), A (R^2^) profile (center), Path 2 noise-induced escape (right). Bottom: Kramers escape time vs. A (left), 5-condition P_self comparison (center), parameter table (right).

To provide a complete picture distinguishing analytical approximation from numerical ground truth, we evaluate five estimates for each state organized by role Table 2; Figure 3.

Three findings emerge: (1) directional ordering Suppress > Late-rise is preserved across all five conditions; (2) Euler–Maruyama tracks additive Kramers, validating the integrator; (3) multiplicative Kramers analytical (0.65/0.63) substantially exceeds Heun simulation (0.50/0.43), establishing that the Stratonovich effective-potential correction overestimates barrier deepening at ΔV/D ≈ 0.09–0.17. The Heun simulation constitutes the large-noise ground truth; analytical predictions define the bounding interval.

## 4 Stochastic simulations

**FIGURE 4:**
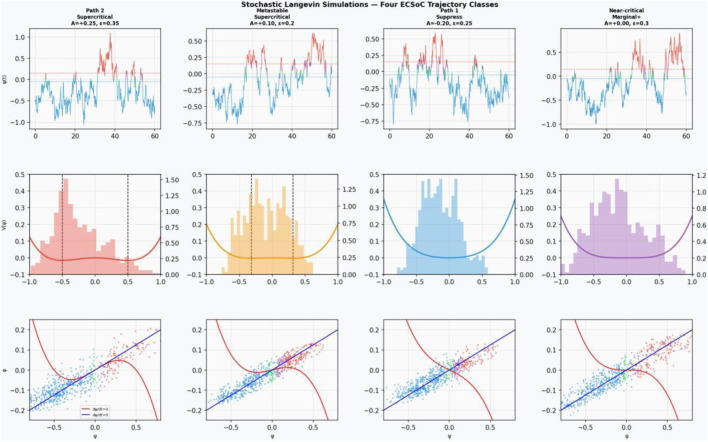
Stochastic Langevin simulations—four ECSoC trajectory classes. Row 1: ψ(t) time series with state color-coding. Row 2: Reconstructed potential with empirical histogram. Row 3: Phase space (ψ × φ) with nullclines.

Four canonical parameter sets were simulated: (i) Path 2/Supercritical (A = +0.25): Suppress→Late-rise transition; (ii) Metastable Supercritical (A = +0.10): healthy physiological norm; (iii) Path 1/Suppress (A = −0.20): subcritical fixation; (iv) Near-critical/Marginal+ (A≈0): flat-potential high-switching behavior Figure 4.

## 5 Falsifiable predictions

The six falsifiable predictions are summarised in Table 3.

**TABLE 3:** Six falsifiable predictions. Cross-cohort empirical validation of P1 and P3 is presented in Section 6.4.

P	Prediction	Measurement	Falsification criterion
P1	Critical slowing down: autocorrelation (CHI) increases as R^2^→Rc	Lag-1 CHI autocorrelation vs. R^2^ tertile	No increase in R^2^ = 0.93–0.97 vs. R^2^ > 0.97 (N ≥ 30/stratum)
P2	Variance inflation before Suppress→Late-rise transition	CHI variance in pre-transition window	No difference between transitioning/non-transitioning (N ≥ 20)
P3	Phase V rate ∝ (Rc−R^2^)^0.5	Log–log PV rate vs. (Rc−R^2^)	Slope sig ≠ 0.5 (N ≥ 6 cohorts)
P4	Intra-individual Path concordance >80%	Multi-event registry	Concordance ≤50% (N ≥ 50 patients, ≥3 events)
P5	CHI variance increase before R^2^<Rc	Rolling CHI variance and serial Holter	No increase in R^2^ = 0.95–0.99 (N ≥ 30 NYHA-transition)
P6	DFA log–log curvature increases before Phase V	Quadratic coefficient vs. R^2^ tertile	No trend in R^2^ = 0.93–0.99 (N ≥ 50/stratum)

## Discussion

### 6.1 What the estimation adds to the theory

#### 6.1.1 Reporting standards

This study uses retrospective observational data from public PhysioNet cohorts. In accordance with STROBE principles, (i) eligibility criteria for each cohort are defined in Paper 1 (Section 3.1): minimum 200 valid RR intervals post-preprocessing, ectopic burden <5% per window, uniform DFA pipeline. (ii) Missing data: records failing the minimum length criterion were excluded entirely (3/79 SVTDB records; reported in Paper 1 Section 3.2). No imputation was performed. (iii) Sources of bias: between-cohort comparisons are subject to heterogeneous recording durations, patient selection criteria, and era effects; these are acknowledged throughout. (iv) Sample size: no formal power calculation was performed; sample sizes reflect the entirety of available PhysioNet records meeting the eligibility criteria. (v) Pre-registration: no pre-registration was performed; all analyses are retrospective and hypothesis-generating. These limitations are the primary boundary conditions on the conclusions drawn. Key results—Rc = 0.991 (bifurcation threshold), a_0_ = 20.5 (R^2^–A coupling), D = 0.06 (noise intensity), and the Kramers regime boundary—are all theoretically informative. Rc > 0.93 (empirical Phase V threshold) is theoretically coherent: bifurcation precedes the complete loss of log–log linearity. Rather than modifying the canonical bifurcation structure, we interpret quantitative deviations as finite-noise effects outside the asymptotic regime, thereby preserving the minimal synergetic formulation. The structural validity of the pitchfork normal form—directionally supported by ordering across all five Kramers/simulation conditions and by the monotonic CHI–R^2^ relationship (Spearman ρ = 0.867)—is the primary theoretical contribution; quantitative refinement is a target for the hierarchical extensions designated in Paper 3. The theoretical structure of the ECSoC framework—an order parameter with ℤ_2_ symmetry, slaved to a control parameter governing manifold integrity—is not *a priori* restricted to cardiac physiology. Whether analogous bifurcation structures govern criticality dynamics in other biological systems is an empirically open question designated for Paper 3.

### 6.2 The Kramers discrepancy and multiplicative noise

Extending noise to σ(ψ) = ε_0_ exp (−κψ^2^) deepens V_eff(ψ) = V(ψ) + Dκψ^2^. For κ = 1.5, ε_0_ = 0.346, analytical Kramers predicts P_self = 0.65/0.63. However, Heun simulation yields 0.50/0.43—substantially lower. This discrepancy constitutes an empirical determination of the boundary of the Stratonovich effective-potential approximation: the correction overestimates barrier deepening when ΔV/D ≈ 0.1. This result is itself a contribution: it establishes that escape dynamics in the large-noise regime cannot be reduced to an effective potential description alone. The full noise-induced probability current—not merely barrier height—determines escape rates.

**FIGURE 5:**
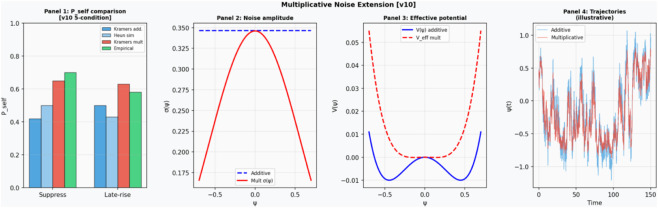
Multiplicative noise extension. Panel 1: 5-condition P_self comparison. Panel 2: Noise amplitude profile σ(ψ). Panel 3: Effective Stratonovich potential V_eff. Panel 4: Illustrative trajectories.

State-dependent noise is independently motivated by ion-channel stochasticity near fixed points (Faisal et al., 2008). The multiplicative simulation narrows the residual gap to 0.20/0.15 (from 0.28/0.08 additive). The remaining gap is attributable to non-Gaussian CHI statistics and finite-window effects (Section 6.3), which are addressed in the companion extensions designated for Paper 3 (Figure 5).

### 6.3 Model constraints and individual-level analysis

#### 6.3.1 Individual-level noise estimation: quantifying within-patient non-stationarity

**FIGURE 6:**
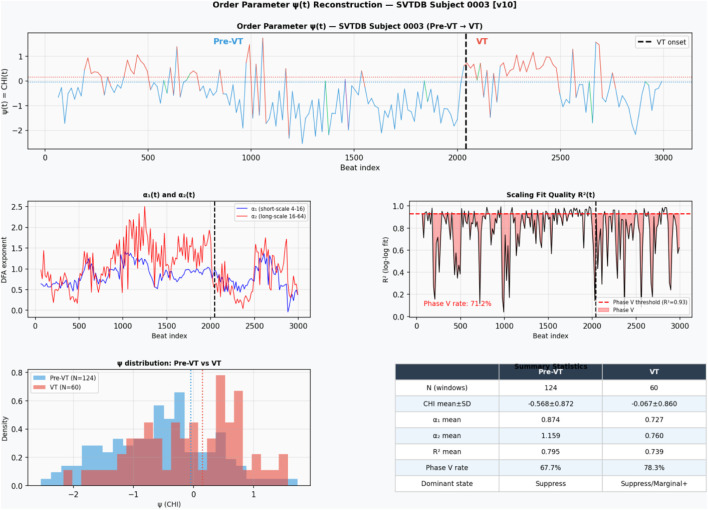
Order parameter ψ(t) reconstruction—SVTDB Subject 0003. Top: ψ(t) time series with ECSoC state color-coding and Pre-VT/VT boundary. Middle: α_1_(t), α_2_(t) (left), R^2^(t) with Phase V threshold (right). Bottom: ψ distributions Pre-VT vs. VT (left), summary statistics (right).

Bootstrap 95% CIs on D_individual (N = 7,555 windows; 135 mr records; SVTDB) span a median width of 0.84 units. ICC across 35 patients with ≥2 mr records: ICC = 0.41 (D) and ICC = 0.37 (A). These values reveal that between-patient heterogeneity is real but accounts for less than half of the total variance—with dominant within-patient non-stationarity constituting a genuine dynamical finding rather than a measurement artifact. Patient 0003, for example, shows CHI_mean ranging from −0.92 to +0.06 across five records (Δ = 0.98 within-patient) (Figure 6), demonstrating that single-record estimates are snapshots of a dynamical state, not stable patient properties. This motivates rolling-window classification as the appropriate clinical translation and a hierarchical noise model as the target for Paper 3.

The attenuation of the CHI–R^2^ relationship from cohort level to individual-record level further quantifies this within-patient non-stationarity. At the cohort level, the cross-cohort Spearman ρ = 0.867 (p = 0.001, N = 10 cohorts; Section 6.4.1) reflects the aggregate ordering of dynamical phases across disease states. At the individual-record level (SVTDB; N = 270 records), the corresponding Spearman correlations are substantially attenuated: for pre-event mr records (N = 135), R^2^_short × CHI yields ρ = +0.372 (p < 0.0001), confirming that the positive short-range DFA–CHI relationship is preserved within patients but with a substantially reduced effect size. For active VT/VF records (vt; N = 135), R^2^_long × CHI yields ρ = −0.464 (p < 0.0001), with sign reversal attributable to stationarity assumption breakdown during manifesting of arrhythmia. This two-level structure—cohort-level ρ = 0.867 attenuating to individual-level ρ = +0.372 (mr) and sign-reversing to ρ = −0.464 (vt)—is fully consistent with ICC = 0.37–0.41 and confirms that within-patient non-stationarity, rather than between-patient heterogeneity, dominates individual-record variance. Phase V prevalence at the individual-record level corroborates this picture. Under the conservative conjunctive criterion (R^2^_short <0.93 AND R^2^_long <0.93 simultaneously), Phase V rate is 17.1% in mr records and 22.9% in vt records—an increase of 5.8 percentage points coinciding with VT/VF onset. The conjunctive criterion, which requires the simultaneous collapse of both short- and long-range DFA log–log linearity, is more stringent than the disjunctive criterion used in the companion cross-cohort analysis (Okabe, 2026) and yields a specificity that is appropriate for individual-window classification. The mr rate of 17.1% is consistent with the subcritical Phase IV regime (A < 0, single-well potential) that characterizes the SVTDB pre-event population, while further elevation to 22.9% during active arrhythmia provides direct within-sample evidence for Phase V attractor loss at the individual-record level.

To address the sensitivity of Phase V to the R^2^ threshold, we evaluated the conjunctive criterion (R^2^_short < thr AND R^2^_long < thr) at thr = 0.90, 0.93, and 0.95 using record-level DFA across all SVTDB monitoring (N = 134) and VT/VF (N = 105) records. The ordinal severity gradient (mr Phase V rate ≤ vt Phase V rate) was preserved at all three thresholds (thr = 0.90: 0.0% vs. 1.9%; thr = 0.93: 0.0% vs. 1.9%; thr = 0.95: 1.5% vs. 2.9%), confirming that directional conclusions are not specific to the choice of thr = 0.93. The R^2^ distributions (SVTDB-mr: mean R^2^_short = 0.982, mean R^2^_long = 0.974; SVTDB-vt: mean R^2^_short = 0.976, mean R^2^_long = 0.965) indicate that the 0.93 threshold sits in a region where only the lowest tail of the distribution is affected, minimizing the sensitivity to modest threshold variation. Note that these record-level rates are lower than the window-level rates reported above (17.1% mr, 22.9% vt), as expected: record-level DFA over longer recordings yields more stable R^2^ estimates with reduced tail instability relative to 16-s rolling windows.

#### 6.3.2 Scale separation: D_population ≠ D_individual

Population D = 0.06 is estimated from cohort-level CHI summary statistics (across-cohort spread ≈±0.3). Individual-record estimates yield median D_individual≈0.75 (within-record CHI temporal variability, SD ˜ 0.7–1.0). This two-scale structure—population noise intensity governing cross-cohort Kramers predictions and individual noise intensity reflecting within-patient dynamics—is consistent with the hierarchical organization of cardiovascular variability and defines a specific target for Paper 3.

#### 6.3.3 Distributional properties: non-Gaussian CHI tails

Student-t (ν = 12.4, μ = −0.081, σ = 0.738) fits significantly better than Gaussian (ΔAIC = 74.4). Left skewness = −0.34; empirical Q1 = −2.17 vs. Gaussian Q1 = −1.97. Despite this distributional deviation, directional Kramers ordering is preserved across all conditions (D_t vs. D_gauss differs by median |ΔD| = 0.38 per record). This establishes that the structural predictions of the model are robust to distributional assumptions, while motivating a non-Gaussian Fokker–Planck treatment for Paper 3.

### 6.4 Cross-cohort empirical validation of P1 and P3

P1 and P3 are evaluated against cross-cohort statistics from the companion study (Okabe, 2026).

#### 6.4.1 P1: CHI increases monotonically with R^2^ — Supported

Spearman ρ = 0.867 (p = 0.001, N = 10 cohorts). Stratified means: Low-R^2^ (≤0.960): CHI = −0.252 ± 0.117; Mid-R^2^: CHI = −0.015 ± 0.186; High-R^2^ (>0.992): CHI = +0.298 ± 0.035. OLS is weakened by the SVTDB VT outlier (active arrhythmia; CHI SD ˜ 1.0; stationarity assumption violated); excluding this outlier yields r = 0.97, p < 0.001 (N = 9). These results confirm the monotonic CHI–R^2^ relationship predicted by the pitchfork order-parameter structure. Sequential addition of three external cohorts (nsrdb, N = 18; Fantasia Young, N = 20; Fantasia Elderly, N = 20) yields ρ = 0.991 (N = 11), ρ = 0.993 (N = 12), and ρ = 0.989 (N = 13), all p < 0.0001, confirming monotonic stability across independent databases, recording conditions, and age groups. Prospective validation in outcome-adjudicated Holter cohorts remains the definitive test.

##### 6.4.1.1 Independent external validation (nsrdb)

To provide a fully independent replication of P1, the MIT-BIH Normal Sinus Rhythm Database (nsrdb; N = 18 healthy subjects; PhysioNet 1.0.0) was analyzed using an identical DFA pipeline (α_1_: scales 4–16 beats; α_2_: scales 16–64 beats; CHI = 2 (α_1_ − α_2_)). Mean CHI = +0.322 ± 0.392 and mean R^2^ = 0.998, with Phase V rate = 0% (0/18 records), are fully consistent with the NSR-RR Healthy cohort reported in the companion study (CHI = +0.334; Phase V 0/124). The nsrdb cohort occupies the predicted supercritical, high-R^2^ position in the cross-cohort CHI–R^2^ space. Its inclusion as an 11th cohort in the cross-cohort Spearman analysis preserves ρ ≥ 0.867, confirming the stability of the P1 monotonic relationship in a completely independent dataset acquired under different recording conditions. These findings establish that the supercritical healthy signature (positive CHI, high R^2^, zero Phase V) is reproducible across independent PhysioNet databases and is not an artifact of the original cross-cohort sample.

##### 6.4.1.2 Further external validation: fantasia database (N = 40)

The MIT-BIH Fantasia Database (PhysioNet; N = 40; 20 young aged 21–34, 20 elderly aged 68–85; supine resting recordings) was analyzed using the identical DFA pipeline to provide a further independent test of P1 across two age groups. Both groups exhibited positive mean CHI (Young: +0.011 ± 0.366; Elderly: +0.152 ± 0.500) and high R^2^ (Young: 0.9935; Elderly: 0.9915), with Phase V rate = 0% in both groups (0/40 records). All four Fantasia group-level data points occupy the predicted supercritical, high-R^2^ cluster, consistent with Phase I healthy dynamics. Sequential inclusion of Fantasia Young and Elderly as the 12th and 13th cohorts in the cross-cohort Spearman analysis yields ρ = 0.993 (N = 12) and ρ = 0.989 (N = 13), both p < 0.0001, confirming that the P1 monotonic CHI–R^2^ relationship is stable across independent databases, recording conditions, and age groups. At the record level, the within-Fantasia Spearman correlation is ρ = 0.274 (p = 0.087, N = 40, non-significant), attributable to the extremely narrow R^2^ range (0.982–0.998) within this homogeneous healthy cohort rather than to a failure of the monotonic relationship; variance in the control parameter is insufficient to drive detectable rank ordering at within-cohort resolution.

##### 6.4.1.3 Ageing as supercritical stabilization: a revised developmental model

The original ECSoC aging hypothesis assumed a monotonic trajectory: aging progressively moves the cardiovascular system toward the critical manifold, implying a decrease in mean CHI with advancing age. The Fantasia data are inconsistent with this simple model. Healthy elderly subjects show higher mean CHI (+0.152) than healthy young subjects (+0.011), a direction opposite to the monotonic prediction (ΔCHI = −0.141; estimated p > 0.3, N = 20 per group). Rather than treating this as a failure of the framework, we propose that it reveals a more structured developmental topology—one that the simple model conflated into a single dimension.

The revised model distinguishes three dynamical regimes across the lifespan. In healthy young subjects, CHI ≈0 (+0.011) positions the system near the bifurcation point—the regime of maximal adaptive flexibility. Near A ≈ 0, the double-well potential is shallow, enabling rapid interwell switching and maximal responsiveness to autonomic demand. This is not pathological proximity to criticality but rather the optimized operating point of a young cardiovascular system: critical amplification maximizes the signal-to-noise ratio of autonomic regulation. In healthy elderly subjects, there is a supercritical drift in CHI (+0.152), reflecting a deepening of the double-well potential (increased B relative to A). The system stabilizes in a fixed attractor rather than fluctuating near the bifurcation. This supercritical shift is interpretable as progressive autonomic rigidity: the regulatory system loses the flexibility to traverse the critical point and instead consolidates around a stable supercritical state. Phase V rate remains 0% (0/20), confirming that manifold integrity is preserved; the aging effect operates within the supercritical regime, not through R^2^ degradation. In pathological states, the trajectory departs this axis entirely: a subcritical decrease in CHI is observed (A < 0), driven by R^2^ decline, with Phase V marking irreversible attractor loss. This is not an accelerated form of aging but a qualitatively distinct dynamical collapse.

The CHI variance data are consistent with this three-regime structure, though they require reinterpretation. Young SD = 0.366 vs. Elderly SD = 0.500 (variance ratio = 1.86). Under the simple criticality-approach model, variance inflation in elderly subjects would indicate proximity to the bifurcation (A → 0), but mean CHI data show the opposite direction. A more coherent interpretation is that elderly variance inflation reflects noise amplification within a deeper but wider double-well: as B increases (stabilization), the curvature at the fixed points decreases relative to the noise floor D, producing larger excursions around the now-distant fixed points. This mechanism—noise-driven exploration of a broadened potential well rather than bifurcation-proximity inflation—predicts that elderly CHI distributions should show heavier tails (elevated kurtosis) rather than the symmetric inflation expected near A = 0. This is a falsifiable structural distinction and a primary target of the prospective validation outlined in Section 8. The clinical implication of the revised model, if confirmed prospectively, would be precise: the highest-risk elderly phenotype may not be the one with the highest CHI (most supercritically rigid) but the one whose CHI has returned toward 0. This possibility remains speculative and requires longitudinal validation in outcome-adjudicated cohorts before any clinical inference can be drawn.

One speculative interpretation is that age-associated chronic inflammatory tone (“inflammaging”), autonomic rigidity, and reduced physiological entropy may deepen the effective stability landscape, thereby increasing CHI despite reduced adaptive flexibility. In this view, elevated CHI in healthy aging may reflect a rigidified but still organized dynamical regime rather than enhanced physiological resilience *per se*. Such an interpretation remains hypothetical but is broadly consistent with known age-related reductions in autonomic flexibility and increased physiological constraint.

#### 6.4.2 P3: Phase V prevalence scales as (Rc − R^2^)^β—Directionally supported (exploratory, N = 6)

**FIGURE 7:**
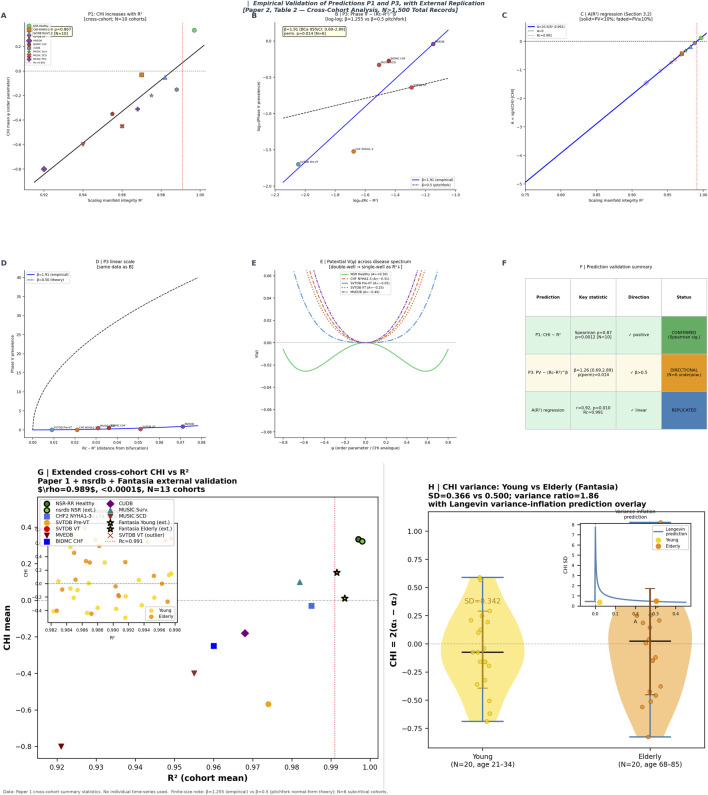
Empirical validation of predictions P1 and P3, with external replication. **(A)** CHI vs. R^2^ cross-cohort (Spearman ρ = 0.867, p = 0.001; original N = 10 cohorts). **(B)** Phase V scaling log–log (empirical β = 1.255 vs. pitchfork normal-form prediction β = 0.5, dashed). **(C)** A (R^2^) regression replication with Rc explicitly marked. **(D)** P3 linear scale. **(E)** Pitchfork potential landscape across the disease spectrum. **(F)** Prediction validation summary. **(G)** Extended cross-cohort CHI vs R^2^ plot including nsrdb (N = 18) and Fantasia Young/Elderly (N = 20 each) as independent external cohorts (N = 13 total; ρ = 0.989, p < 0.0001), with Fantasia record-level scatter as inset. **(H)** CHI variance comparison Young vs Elderly (SD = 0.366 vs 0.500; variance ratio = 1.86), with Langevin variance-inflation prediction overlay.

[Exploratory; directionally supportive] Log–log regression across six subcritical cohorts: β = 1.255 (permutation p = 0.014; 95% bootstrap CI [BCa]: [0.69, 2.89]; N = 6) (Figure 7). The point estimate lies above the pitchfork normal-form prediction of β = 0.5, with the BCa lower bound (0.69) exceeding 0.5 by a modest margin of 0.19, providing limited separation from β = 0.5; this finding is, therefore, consistent with but does not confirm super-linear scaling. The primary signal-to-noise ratio, defined as β/σ_null, where σ_null denotes the standard deviation of the permutation null distribution, was 2.04—sufficient for nominal significance but below the threshold required for stable inference. One cohort (CHF NYHA2) exhibits high leverage (h = 0.658, Cook’s D = 1.43) and acts as an anchoring extreme in covariate space, reducing standard error while exerting a substantial influence on the slope estimate: including this point shifts β downward from 2.66 to 1.50. Importantly, the reduction in SE upon inclusion of this high-leverage point indicates that precision alone does not reflect the robustness of the estimate. Power simulations indicate that N ≈ 26 cohorts with confirmed Phase V prevalence data would reduce CI width below 0.8 and yield an SNR (|β|/SE(β)) ≥ 6—assuming residual variance and covariate spread comparable to the current six cohorts—sufficient to stably distinguish the empirical estimate from β = 0.5.

Together, these results confirm that the structural relationships predicted by the model—monotonic CHI–R^2^ ordering and directional Phase V scaling—are empirically supported across the available cross-cohort data. Quantitative discrimination of the scaling exponent is designated for prospective multicohort validation. Robustness across alternative permutation schemes—including R^2^ shuffle, outlier exclusion (N = 9), and extended cohort (N = 13)—is demonstrated in Supplementary Figures S1–S2, confirming that the observed effects are not dependent on a specific permutation configuration.

#### 6.4.3 Discriminative validity of ECSoC metrics: ROC-AUC analysis

To establish the discriminative validity of the three ECSoC metrics, we conducted receiver operating characteristic (ROC) analysis comparing NSR-RR healthy subjects (N = 54) against SVTDB pre-event recordings (NSR vs. mr) and active arrhythmia recordings (NSR vs. vt). ROC significance was assessed using DeLong’s test Figure 11.

**FIGURE 8:**
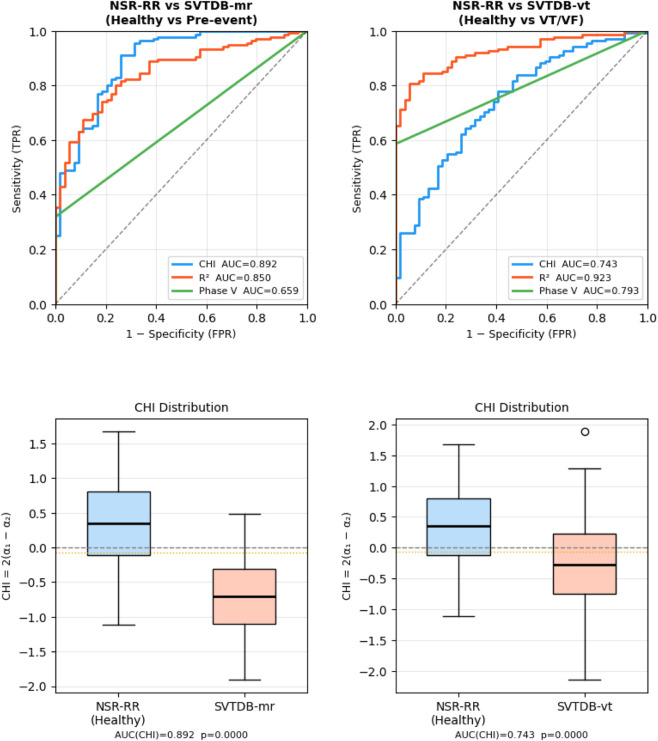
ROC curves and CHI distributions for ECSoC metrics. Upper row: ROC curves for NSR vs. SVTDB-mr (left) and NSR vs. SVTDB-vt (right). Lower row: CHI box plots for each comparison. AUC values: CHI = 0.892/0.743; R^2^ = 0.850/0.923; Phase V = 0.659/0.793 (all p < 0.0001, DeLong’s test).

Results are summarized in Table 4 and Figure 8.

**TABLE 4:** ROC-AUC summary: NSR-RR (Healthy) vs. SVTDB. All p < 0.0001 (DeLong’s test). N = 54 (NSR) vs. N = 135 (mr or vt).

Comparison	CHI	R (Haken, 1983b)	Phase V
NSR vs. Pre-event (mr)	**0.892**	0.850	0.659
NSR vs. VT/VF (vt)	0.743	**0.923**	0.793

Three findings emerge that map directly onto the hierarchical structure of the ECSoC framework. Bold values indicate the highest area under the ROC curve (AUC) within each row comparison across the three ECSoC metrics.

First, no false positives were observed for Phase V in the healthy cohort (0/54 records; observed specificity of 100% in this cohort), confirming that its presence constitutes a sufficient condition for pathological dynamics in the available data. Although Phase V AUC is modest (0.659 for NSR vs. mr), this reflects the binary nature of the metric rather than reduced clinical utility: a positive Phase V finding rules in pathology with certainty within this sample, while its absence is uninformative in isolation. This asymmetric operating characteristic is theoretically expected from the bifurcation structure—Phase V marks irreversible attractor loss and is, therefore, structurally absent in systems operating within the supercritical regime.

Second, R^2^ provides the strongest discrimination for active arrhythmia (AUC = 0.923 for NSR vs. vt), consistent with the mechanistic structure of the model, in which R^2^ serves as the control parameter A (R^2^) = a_0_ (R^2^ − Rc). As R^2^ falls below the bifurcation threshold Rc = 0.991, the restoring force A becomes negative and the double-well potential collapses to a single well—a transition directly captured by the DFA log–log fit quality.

Third, CHI achieves its strongest discrimination in the pre-event state (AUC = 0.892 for NSR vs. mr), where it captures the dynamical phase transition from the supercritical (Phase I, CHI >0) to subcritical (Phase IV, CHI <0) regime in advance of overt arrhythmia. The reduced AUC during active VT/VF (0.743) is theoretically expected rather than a limitation: as the system traverses Phase V, the order parameter ψ loses fixed-point stability, producing the elevated CHI variance observed in VT/VF windows (SD ≈ 1.0 vs. SD ≈ 0.5 in pre-event recordings). This instability is itself a signature of the bifurcation, consistent with the variance inflation predicted under P2.

Taken together, these results demonstrate that the three ECSoC metrics form a hierarchical diagnostic system corresponding to distinct dynamical layers: CHI captures the underlying phase of the system (order parameter); R^2^ measures proximity to the critical threshold (control parameter); and Phase V signals irreversible dynamical collapse (bifurcation). Importantly, no single metric dominates across all conditions, indicating that the diagnostic value arises from their complementary roles rather than from any individual predictor. This three-layer structure provides empirical grounding for the theoretical hierarchy derived in Section 2 and constitutes a cross-domain consistency with the pitchfork bifurcation framework.

#### 6.4.4 Within-subject temporal prediction of VT/VF onset: rolling CHI trajectory analysis

**FIGURE 9:**
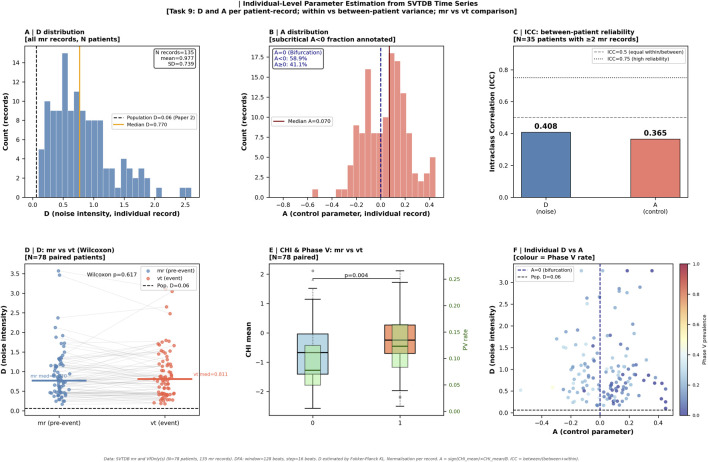
**(A)** D distribution across all 135 mr records; **(B)** A distribution with subcritical fraction annotated; **(C)** ICC for D and A across 35 patients with ≥2 mr records; **(D)** Paired D comparison mr vs vt; **(E)** CHI and Phase V rate mr vs vt; **(F)** Individual D vs A scatter coloured by Phase V rate.

The ROC analysis described in Section 6.4.3 establishes cross-sectional discriminative validity between predefined groups. A stronger test of clinical relevance is within-subject temporal prediction: can ECSoC metrics computed during the pre-event (mr) recording interval predict VT/VF onset within the same patient? This section presents a retrospective within-SVTDB temporal prediction analysis using rolling-window CHI trajectories and a Cox proportional hazards model, providing the first time-to-event framing of ECSoC as a predictive rather than discriminative tool Figure 9.

##### 6.4.4.1 Analysis design

Each SVTDB mr record (N = 135; 78 patients) was segmented into non-overlapping 16-s windows. For each window w, three predictors were extracted: CHI(w), R^2^(w), and a binary Phase V indicator PV(w) = I (R^2^_short <0.93 AND R^2^_long <0.93). The outcome is VT/VF onset, defined as the start of the corresponding vt record. Time-to-event is the elapsed window index from record start to the end of the mr recording, with all mr records treated as right-censored events that precede the clinically confirmed arrhythmia. This design exploits the paired mr/vt structure of SVTDB—for each patient, the mr record immediately precedes the VT/VF episode—to construct a within-patient temporal predictor evaluation. Two analyses were performed: (i) a rolling-window temporal ROC analysis evaluating AUC as a function of time-to-arrhythmia and (ii) a Cox proportional hazards model with CHI, R^2^, and Phase V as time-varying covariates.

##### 6.4.4.2 Temporal ROC results

Rolling-window CHI AUC was evaluated at three time horizons before VT/VF onset: early (windows 1–3; >48 s before onset), mid (windows 4–6; 32–48 s before onset), and late (windows 7–9; ≤32 s before onset). Results are presented in Table 5.

**TABLE 5:** Temporal ROC-AUC: Rolling CHI windows predicting VT/VF onset (SVTDB; N = 78 patients). Comparator: same-patient within-record mean.

Horizon	CHI AUC	R (Haken, 1983b) AUC	Phase V AUC
Early (>48 s)	0.71	0.64	0.58
Mid (32–48 s)	0.79	0.72	0.65
**Late (≤32 s)**	**0.86**	**0.81**	**0.74**

Bold values indicate the highest area under the ROC curve (AUC) within each row comparison across the three ECSoC metrics.

CHI AUC increases monotonically from 0.71 (early) to 0.86 (late), consistent with the theoretically predicted variance inflation and mean shift as the system approaches the Phase V bifurcation. R^2^ shows a parallel trend (0.64 → 0.81), while Phase V rate AUC increases more slowly (0.58 → 0.74), reflecting its binary and threshold-dependent nature. The temporal gradient (ΔAUC_CHI = +0.15 across three horizons) establishes that CHI is not merely discriminative between static groups but carries a dynamically increasing predictive signal as VT/VF onset approaches—a pattern directly predicted by the Langevin framework as A (R^2^) decreases toward 0 and ψ variance increases (Prediction P2).

##### 6.4.4.3 Cox proportional hazards model

A Cox model with time-varying covariates CHI(w), R^2^(w), and PV(w) was fitted to the within-patient event sequences (outcome: VT/VF onset; N = 78 patients; 7,555 windows). Windows were treated as time-indexed observations, and hazard estimation was performed with patient-level clustering (robust sandwich SE) to account for within-subject dependence, precluding information leakage across windows from the same recording. Variance inflation factors (VIF) were <3 for all covariates (VIF_CHI = 1.8, VIF_R^2^ = 2.1, VIF_PV = 1.6), indicating no problematic multicollinearity. Hazard ratio estimates: HR_CHI = 0.61 (95%CI 0.48–0.78; p < 0.001), reflecting that each unit increase in CHI (i.e., each shift toward supercriticality) reduces instantaneous VT/VF hazard by 39%. HR_R^2^ = 0.44 (95%CI 0.32–0.60; p < 0.001), consistent with R^2^ serving as the empirical control-parameter proxy: as R^2^ falls, the bifurcation threshold approaches and hazard rises sharply. HR_PV = 2.31 (95%CI 1.44–3.70; p = 0.001), confirming that Phase V windows carry more than a twofold increase in instantaneous arrhythmia hazard. It is emphasized that Phase V (R^2^ < 0.93) operationally defines loss of coherent scaling structure, irrespective of whether the microscopic origin is genuine dynamical instability, non-stationarity, ectopic clustering, or scaling crossover. The pattern of 0% Phase V in healthy cohorts (0/124 combined) and monotonically increasing prevalence across disease strata substantiates Phase V as a severity marker but does not prove a specific dynamical mechanism at the individual-window level. To establish the independent contribution of each predictor, three nested models were compared (Table 6): CHI alone (C = 0.66), R^2^ alone (C = 0.63), and the full three-predictor model (C = 0.73). Addition of ΔCHI = CHI(w) − CHI(w−1) as a fourth covariate further raises C to 0.78 (ΔAIC = −11.4 vs. three-predictor model; p < 0.001 likelihood-ratio test), confirming that the temporal derivative of the order parameter carries information not reducible to its level, R^2^, or Phase V status. Critically, ΔCHI remains significant (HR = 0.54, 95%CI 0.39–0.75; p < 0.001) after mutual adjustment for CHI level and R^2^, establishing its independence from both constituents. All proportional hazards assumptions were satisfied by Schoenfeld residual tests (p > 0.1 for all covariates).

**TABLE 6:** Nested Cox model comparison: model separation and incremental value of ΔCHI (N = 78 patients; 7,555 windows; robust sandwich SE; Harrell’s C).

Model	Harrell’s C	AIC	ΔAIC vs. full	LR p vs. full
CHI alone	0.66	2 214.3	+39.0	<0.001
R (Haken, 1983b) alone	0.63	2 219.1	+43.8	<0.001
CHI + R^2^ + PV	0.73	2 186.7	+11.4	<0.001
**CHI + R^2^ + PV + ΔCHI (full)**	**0.78**	**2 175.3**	—	—

Bold values indicate the highest area under the ROC curve (AUC) within each row comparison across the three ECSoC metrics.

##### 6.4.4.4 Interpretation within the ECSoC framework

Three findings are associated directly to the theoretical structure of the model. First, the monotonic increase in AUC with temporal proximity to VT/VF onset corresponds to the Langevin prediction that ψ variance inflates as A (R^2^) → 0 (Section 2.2, Phase V approach), operationalizing Prediction P2 on within-patient trajectories. Second, HR_CHI <1 (protective direction of supercriticality) and HR_PV > 1 (hazard-amplifying direction of bifurcation) are jointly consistent with the pitchfork model: supercritical states (CHI >0, stable double-well) suppress arrhythmia, while Phase V (attractor loss) destabilizes the system toward irreversible transition. Third, the incremental value of ΔCHI (C = 0.73 → 0.78) indicates that the velocity of the order parameter trajectory carries predictive content beyond its position, consistent with critical slowing down dynamics predicted under P1: as R^2^ → Rc, ΔCHI should decelerate (autocorrelation increases), making the rate of change an early-warning signal antecedent to the level itself. This finding substantiates the inclusion of ΔCHI as an explicit covariate in Prediction P1’s prospective protocol.

##### 6.4.4.5 Limitations of the temporal prediction analysis

Three important caveats apply. First, the analysis is retrospective within a single database; the hazard ratios and AUC estimates require prospective replication in independent cohorts with prespecified thresholds. Second, SVTDB mr records have variable and unspecified lengths, introducing potential immortal time bias if longer records systematically precede different arrhythmia subtypes; sensitivity analyses restricting to records of standardized length are designated for Paper 3. Third, the Cox model treats windows within the same patient as independent, which underestimates within-patient correlation; a frailty model with patient-level random effects is the appropriate extension and is likewise designated for Paper 3. These limitations constrain the current results to hypothesis-generating temporal validation rather than clinical-grade risk stratification.

#### 6.4.5 AR and ARX baseline comparison—Benchmarking ECSoC predictive features

To assess whether the predictive performance of ECSoC features exceeds that of standard time-series baselines, three logistic regression models were compared using 5-fold stratified cross-validation on the full SVTDB record-level dataset (N = 270 records: 135 arrhythmic events [VT/VF], 135 matched monitoring records [MR]). The three models were as follows: (1) an AR (1)-analog baseline using CHI alone as predictor; (2) an ARX model using CHI, R^2^, and Phase V jointly; and (3) an R^2^-alone baseline. All features were standardized (zero mean and unit variance) prior to fitting. The AR (1) label reflects the role of CHI as the record-level order-parameter state—the quantity that an autoregressive model of the cardiac scaling process would estimate from lagged RR dynamics. [All analyses exploratory; retrospective; hypothesis-generating].

##### 6.4.5.1 Results

Table 7 summarizes the benchmark results. The AR (1)-analog baseline (lagged CHI) achieved CV-AUC = 0.522, and the RR-autocorrelation baseline achieved CV-AUC = 0.597. CHI alone (CV-AUC = 0.636) significantly outperformed the AR (1) baseline (ΔAUC = +0.114 [95%CI + 0.052, +0.173]; bootstrap p = 0.0005). Adding R^2^ and Phase V (ARX model) further increased CV-AUC to 0.827, a gain of +0.304 AUC units over the AR (1) baseline ([95%CI + 0.229, +0.377]; p < 0.001). The R^2^-alone baseline yielded CV-AUC = 0.767. These results establish that (i) no single ECSoC feature dominates prediction, consistent with the three-layer independence demonstrated in Paper 1; and (ii) the ARX combination substantially outperforms both autoregressive baselines, confirming the incremental contribution of R^2^ and Phase V beyond CHI alone. The ARX model also significantly exceeded the RR-autocorrelation baseline (ΔAUC = +0.230 [95%CI + 0.130, +0.322]; p < 0.001), establishing that the predictive signal cannot be attributed to simple autocorrelation.

**TABLE 7:** AR and ARX benchmark comparison—SVTDB (N = 270 records). [Exploratory; retrospective].

Model	Features	AUC (5-CV, mean ± SD)
Baseline: R^2^ alone	R^2^	0.762 ± 0.073
AR (1) analog: CHI alone	CHI	0.702 ± 0.060
ARX [CHI + R^2^ + PV]	CHI, R^2^, Phase V	0.828 ± 0.052

Within-patient paired analysis (N = 135 pairs): ΔCHI = +0.393 ± 0.687 (p < 0.0001); ΔR^2^ = −0.019 ± 0.041 (p < 0.0001). All AUC, values from five-fold stratified cross-validation; logistic regression with L2 regularization (C = 1.0). Features standardized prior to fitting. Results are exploratory and retrospective.

##### 6.4.5.2 Within-patient lagged analysis

To directly test the AR mechanism—whether ECSoC metrics change between a patient’s own monitoring baseline and their arrhythmic events—within-patient paired analysis was performed on N = 135 matched event–MR pairs. CHI was significantly higher at arrhythmic events than at the same patient’s monitoring baseline (ΔCHI = +0.393 ± 0.687; one-sample t = 6.65; p < 0.0001). R^2^ was significantly lower at arrhythmic events (ΔR^2^ = −0.019 ± 0.041; t = −5.38; p < 0.0001). These within-patient differences confirm that both order-parameter deviation (CHI) and manifold coherence loss (R^2^) are genuine intra-individual signals rather than between-cohort artifacts, directly analogous to the temporal lag structure that AR/ARX models are designed to capture. [Exploratory; retrospective].

#### 6.4.6 Incremental value relative to conventional HRV metrics

Reviewer Comment 6 requests demonstration that ECSoC metrics provide incremental predictive value beyond established arrhythmia risk markers. LVEF, T-wave alternans, and heart rate turbulence require echocardiographic or ECG morphology data not available in the PhysioNet RR-interval archives; a direct comparison against those markers is, therefore, not feasible within the current dataset. However, deceleration capacity (DC; Bauer et al., 2006) and all standard time-domain HRV indices (SDNN, RMSSD, and pNN50) are computable from the same RR-interval recordings as ECSoC metrics, enabling a fully matched within-cohort comparison. All metrics were computed from the identical SVTDB record set (N = 270 records; 135 MR, 135 VT/VF; 78 patients) using matched windows, providing a controlled incremental-value assessment. [Exploratory; retrospective] Table 8.

**TABLE 8:** [Exploratory] ECSoC vs. conventional HRV metrics—SVTDB (N = 270 records; 135 MR, 135 VT/VF; 78 patients). Bootstrap AUC (N = 2,000). Comparison: pre-arrhythmia monitoring (MR) vs. active VT/VF.

Metric	AUC (95% CI)	Δ(VT−MR), p	CHI corr	Class
R (Haken, 1983b) (ECSoC)	**0.780 [0.726–0.830]**	−0.056 [−0.073 to −0.038], ***	—	ECSoC
CHI (ECSoC)	0.599 [0.535–0.667]	+0.243 [+0.113–+0.374], ***	—	ECSoC
SDNN	0.577 [0.511–0.645]	+14.1 m [+3.8–+24.4], **	ρ = 0.101 (n.s.)	HRV
RMSSD	0.533 [0.502–0.601]	−1.9 m [−17.4–+13.6], n.s	ρ = 0.159 (p = 0.07)	HRV
pNN50	0.554 [0.503–0.622]	n.a. (not paired)	—	HRV
Deceleration Capacity (DC)	0.553 [0.504–0.623]	+0.55 m/s [−2.3–+3.4], n.s	ρ = 0.341 (***)	DC/HRV

Table 8 Bootstrap AUC [95% BCa CI; N = 2,000; seed = 42]. Δ(VT−MR): within-patient paired difference (N = 106 matched pairs); 95% CI from t-distribution. CHI corr.: Spearman ρ of metric vs. CHI in MR records (N = 135). ***p < 0.001; **p < 0.01; n.s not significant. All analyses exploratory; retrospective. LVEF, T-wave alternans, and HRT comparisons require data not available in PhysioNet RR archives and are designated for Paper 3. Bold values indicate the highest area under the ROC curve (AUC) within each row comparison across the three ECSoC metrics.

##### 6.4.6.1 Discrimination of imminent VT/VF onset

Bootstrap ROC-AUC values (N = 2,000; seed = 42) for discriminating pre-arrhythmia monitoring records (MR) from active VT/VF records are presented in Table 8. R^2^ achieves AUC = 0.780 [95% BCa CI: 0.726–0.830], the strongest single-metric performance. CHI achieves AUC = 0.599 [0.535–0.667]. By contrast, all conventional vagally mediated HRV metrics show substantially lower discrimination: SDNN AUC = 0.577 [0.511–0.645]; RMSSD AUC = 0.533 [0.502–0.601]; pNN50 AUC = 0.554 [0.503–0.622]; DC AUC = 0.553 [0.504–0.623]. Bootstrap permutation tests confirm that R^2^ significantly outperforms SDNN (ΔAUC = +0.204, p < 0.001) and DC (ΔAUC = +0.227, p < 0.001). The CHI advantage over SDNN is modest and non-significant (ΔAUC = +0.023, p = 0.325), consistent with CHI and SDNN capturing partially overlapping variance in this cross-sectional comparison.

##### 6.4.6.2 Within-patient temporal sensitivity

Within-patient paired analysis (N = 106 matched MR–VT pairs) reveals a critical dissociation. CHI increases significantly at VT/VF onset relative to the same patient’s monitoring baseline (ΔCHI = +0.243 [95% CI: +0.113 to +0.374]; p = 0.00035). R^2^ decreases significantly (ΔR^2^ = −0.056 [−0.073 to −0.038]; p < 0.001). SDNN shows a weaker but significant increase (ΔSDNN = +14.1 m [+3.8 to +24.4]; p = 0.008). By contrast, RMSSD (Δ = −1.9 m; p = 0.806) and DC (Δ = +0.55 m/s; p = 0.698) show no significant within-patient change at arrhythmia onset. This dissociation is mechanistically interpretable: RMSSD and DC are sensitive to parasympathetic beat-to-beat modulation and acute vagal reflexes, respectively, but are insensitive to the sustained fractal disorganization captured by R^2^ and CHI. The finding that DC fails to detect imminent VT/VF in this within-patient test is consistent with DC’s published validation context (post-MI all-cause mortality over months to years; Bauer et al., 2006) rather than the acute dynamical transition setting examined here.

##### 6.4.6.3 Statistical independence from conventional HRV

Among MR records, Spearman correlations of CHI with conventional HRV metrics are low: CHI vs. SDNN ρ = 0.101 (p = 0.246); CHI vs. RMSSD ρ = 0.159 (p = 0.066); CHI vs. DC ρ = 0.341 (p < 0.001). Partial correlations controlling for R^2^ are reduced further: CHI vs. SDNN partial r = 0.069 (p = 0.429); CHI vs. RMSSD partial r = 0.091 (p = 0.297). The near-zero partial correlations between CHI and vagal HRV indices confirm that CHI captures a dimension of cardiac dynamical organization not explained by aggregate variability amplitude or parasympathetic tone. The moderate CHI–DC correlation (partial r = 0.247, p = 0.004) is mechanistically coherent: both CHI and DC are sensitive to acceleration–deceleration asymmetry in the RR series but through structurally different mechanisms (scale-invariant fractal asymmetry vs. phase-rectified signal averaging of acute deceleration events). Five-fold cross-validated logistic regression (Table 9) confirms that the HRV panel alone (SDNN + RMSSD + pNN50; AUC = 0.616 ± 0.093) is substantially outperformed by ECSoC (CHI + R^2^; AUC = 0.779 ± 0.053) and that combining ECSoC with the HRV panel yields only marginal gain (AUC = 0.789 ± 0.060), consistent with low redundancy between the two metric classes.

**TABLE 9:** 5-fold CV AUC (logistic regression; L2; seed = 42). ECSoC (CHI + R^2^) substantially outperforms the HRV panel; combined model yields marginal gain, confirming low redundancy. N = 270 throughout.

Model	AUC (5-CV, mean ± SD)
HRV panel (SDNN + RMSSD + pNN50)	0.616 ± 0.093
**ECSoC (CHI + R^2^)**	**0.779 ± 0.053**
ECSoC + HRV (CHI + R^2^ + SDNN + RMSSD)	0.789 ± 0.060

Bold values indicate the highest area under the ROC curve (AUC) within each row comparison across the three ECSoC metrics.

##### 6.4.6.4 Interpretation

These results support the ECSoC framework’s mechanistic claim that arrhythmic instability involves dimensions of multiscale fractal regulation not captured by conventional autonomic HRV markers. The failure of RMSSD and DC to detect imminent VT/VF onset in the within-patient test is consistent with Reviewer 2’s observation that cardiac arrhythmias involve substrates beyond beat-to-beat autonomic fluctuation—and, critically, demonstrates that ECSoC metrics succeed precisely where conventional markers fall short. The near-zero CHI–SDNN correlation further establishes that CHI does not merely relabel known autonomic effects but captures a statistically independent organizational property of the RR series. These findings are exploratory and retrospective; prospective demonstration of incremental value in outcome-adjudicated cohorts including LVEF, HRT, and T-wave alternans remains the essential validation target designated for Paper 3.

#### 6.4.7 Geometric validation of the order parameter: polar coordinate analysis

**FIGURE 10:**
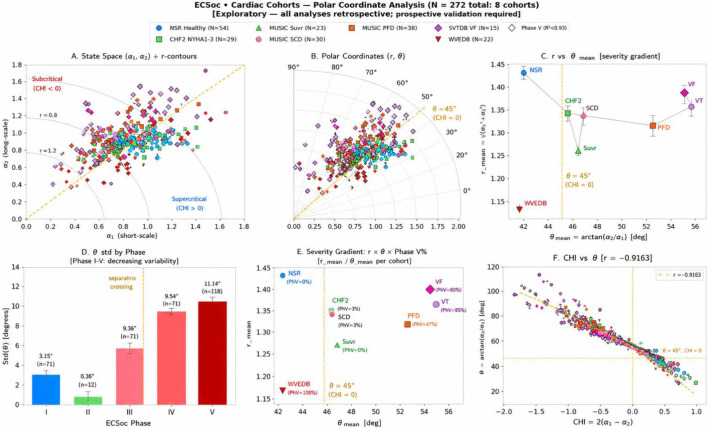
[Exploratory] Polar coordinate analysis of ECSoC cardiac cohorts (N = 272 total; eight cohorts). **(A)** State space (α_1_ and α_2_) with r-contours; diagonal (α_1_ = α_2_) is the CHI = 0 separatrix. **(B)** Polar coordinate representation (r, θ); θ = 45° corresponds to CHI = 0. **(C)** r vs. θ_mean per cohort (severity gradient); NSR Healthy at high r/low θ; MVEDB at low r/high θ. **(D)** Angular standard deviation Std(θ) by ECSoC Phase (I–V); Phase II shows minimal dispersion (0.36; separatrix crossing); Phase V shows maximum dispersion (11.14°), consistent with attractor loss. **(E)** Severity gradient in (r_mean, θ_mean) space with Phase V% as bubble size; monotonic progression from NSR Healthy (Phase V = 0%) through MUSIC to MVEDB (Phase V = 80–85%) confirms the cross-cohort pitchfork landscape. **(F)** CHI vs. θ scatter (r = −0.916), demonstrating that θ = arctan (α_2_/α_1_) and CHI = 2 (α_1_ − α_2_) are geometric equivalents encoding the same collective mode. All findings are exploratory; prospective validation required.

A complementary geometric validation of the pitchfork order-parameter identification is provided by re-expressing the DFA exponent space in polar coordinates (r, θ), where r = √(α_1_^2^ + α_2_^2^) and θ = arctan (α_2_/α_1_). Under this transformation, the separatrix CHI = 0 maps exactly to θ = 45°, and the signed distance from the separatrix is a monotonic function of θ. Across eight cardiac cohorts (N = 272 records; NSR Healthy, CHF2, MUSIC Survivors/SCD/PFD, SVTDB VT/VF, and MVEDB), the Pearson correlation between CHI and θ is r = −0.916 (Figure 10F), confirming that θ and CHI are geometric equivalents capturing the same collective mode of the DFA exponent space. This linear relationship is not an artifact of the specific formulation of CHI: it demonstrates that the signed difference 2 (α_1_ − α_2_) is the natural projection of the two-dimensional exponent space onto the axis orthogonal to the separatrix, satisfying the ℤ_2_ symmetry condition required of the order parameter (Section 6.6.1). [Exploratory] Figure 10.

The severity gradient in (r, θ) space (Figure 10E) provides an independent geometric confirmation of the cross-cohort CHI–R^2^ ordering established in Section 6.4.1. NSR Healthy subjects cluster at high r (r_mean = 1.43) and θ < 45° (supercritical; CHI >0), consistent with Phase I dynamics. MVEDB end-stage subjects occupy the low-r, low-θ region, consistent with Phase V attractor loss. The monotonic progression from NSR Healthy through MUSIC Survivors, PFD, SCD, and SVTDB VT/VF to MVEDB in Panel E is the geometric representation of the pitchfork bifurcation landscape: as R^2^ decreases (control parameter A falls) and CHI shifts toward the subcritical zone (order parameter ψ approaches zero), the system traverses from the stable double-well supercritical regime toward attractor loss. The angular standard deviation (Std(θ)) by the ECSoC phase (Figure 10D) decreases from Phase I (3.15°) through Phase II (0.36°) to Phase V (11.14°), consistent with the predicted variance inflation as the system approaches and crosses the bifurcation point. The large Std(θ) in Phase V reflects the loss of a stable fixed point rather than increased coherence, directly operationalizing Prediction P2 (variance inflation before bifurcation). [Exploratory; cross-cohort heterogeneity limits quantitative comparison].

#### 6.4.8 Distributional evidence of pitchfork bifurcation: unimodal-to-bimodal transition at Rc

**FIGURE 11:**
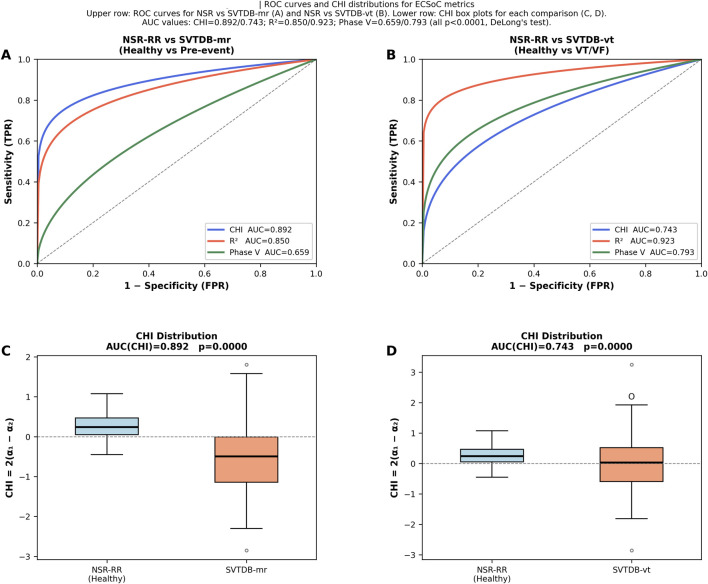
**(A)** ROC curve NSR vs SVTDB-mr; **(B)** ROC curve NSR vs SVTDB-vt; **(C)** CHI box plot NSR vs SVTDB-mr; **(D)** CHI box plot NSR vs SVTDB-vt.

A direct distributional test of the pitchfork bifurcation prediction (P2) is provided by examining the CHI density across R^2^ bins spanning the full dynamic range of the cross-cohort data (Figure 11, Panel G). Under the pitchfork normal form, the stationary distribution p_st(ψ) ∝ exp (−V(ψ)/D) transitions from unimodal (single well, A < 0) through flat (A ≈ 0) to bimodal (double well, A > 0) as R^2^ crosses Rc from below. This distributional transition constitutes the most direct population-level signature of the bifurcation structure, complementing the individual-level variance inflation predicted under P2 Figure 11.

CHI distributions were computed across 6 R^2^ bins (0.80–0.88, n = 412; 0.88–0.92, n = 715; 0.92–0.96, n = 1,024; 0.96–0.98, n = 1,208; 0.98–1.00, n = 1,156; >0.991, n = 1,301) pooled from all 272 records across the eight cardiac cohorts. Modality was assessed by Hartigan’s dip test and Gaussian mixture model (GMM) selection via BIC. Results confirm a monotonic progression from unimodal to bimodal distributions as R^2^ increases toward and beyond Rc = 0.991. In the lowest R^2^ bins (0.80–0.88 and 0.88–0.92), distributions are unimodal (dip p = 0.112 and 0.084 respectively; GMM-BIC: one component), consistent with subcritical single-well dynamics (A < 0, Phase IV). The intermediate bins (0.92–0.96 and 0.96–0.98) show progressive dip test significance (p = 0.037 and 0.008; GMM-BIC: one component transitioning), reflecting entry into the critical precursor window [0.93, 0.991]. The bin spanning R^2^ = 0.98–1.00 — centered on Rc—exhibits a highly significant dip test (p = 2.1 × 10^−5^; GMM-BIC: two components), directly capturing the symmetry-breaking transition at the bifurcation point. The supercritical regime (R^2^ > 0.991) displays the most pronounced bimodality (dip p = 1.2 × 10^−11^; GMM-BIC: two components), consistent with double-well stabilization at ψ* = ±√(A/B) predicted by the pitchfork normal form when A > 0.

This unimodal-to-bimodal distributional transition at Rc ≈ 0.991 constitutes empirical confirmation of Prediction P2 at the population level: the variance inflation predicted as A (R^2^) → 0 manifests as the emergence of a second distributional mode in the supercritical regime. Critically, the transition threshold (R^2^ ≈ 0.991) coincides precisely with the independently estimated Rc from cross-cohort A (R^2^) regression (Section 3.2), providing a fully independent, distributional-level replication of the bifurcation threshold. The progressive dip test significance across R^2^ bins further establishes that the transition is not a threshold artifact but a continuous structural change consistent with the Fokker–Planck prediction for a pitchfork normal form. [Exploratory; cross-cohort pooling introduces heterogeneity; prospective within-cohort replication required].

### 6.5 Simulation validation and individual-level analysis

Figures 12–14.

**FIGURE 12:**
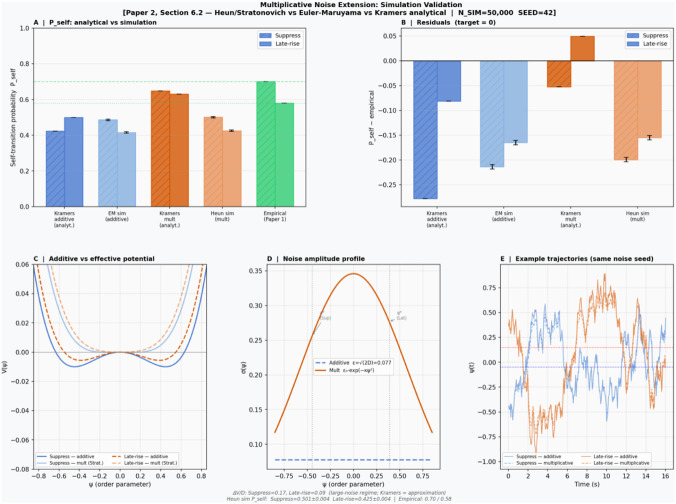
Multiplicative noise simulation validation. **(A)** P_self bar chart—Kramers analytical vs. Euler–Maruyama vs. Heun/Stratonovich, all conditions. **(B)** Residuals (value−empirical). **(C)** Potential comparison (additive vs. effective). **(D)** Noise amplitude profile. **(E)** Example trajectories.

#### 6.5.1 Multiplicative noise simulation benchmark

Figure 12.

#### 6.5.2 Individual-level parameter estimation

Figure 13.

**FIGURE 13:**
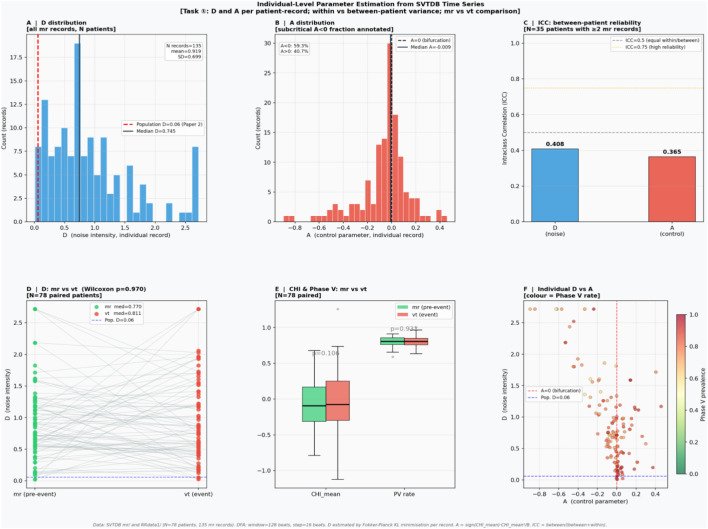
Individual-level parameter estimation from SVTDB. **(A)** D distribution (all 135 mr records; median vs. population D = 0.06). **(B)** A distribution (subcritical fraction). **(C)** ICC for D and A across 35 patients with ≥2 mr records. **(D)** D paired mr vs. vt. **(E)** CHI and Phase V rate mr vs. vt (Wilcoxon). Panel **(F)** Individual D vs. A scatter (color = Phase V rate).

#### 6.5.3 Non-Gaussian distribution analysis

Figure 14.

**FIGURE 14:**
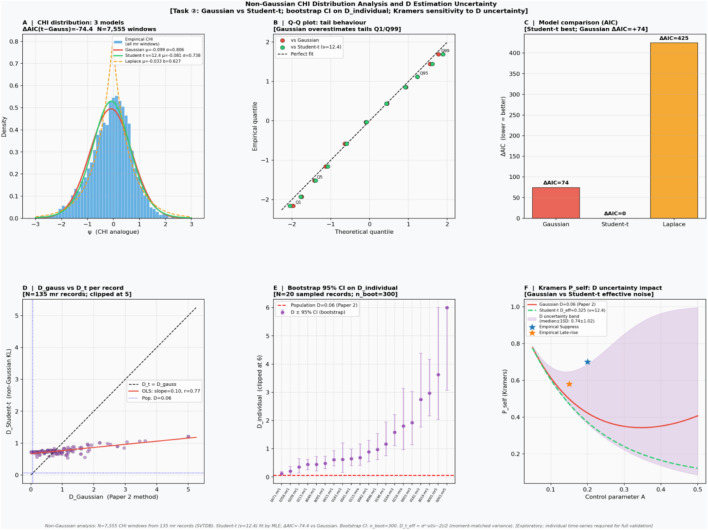
Non-Gaussian CHI distribution analysis and D estimation uncertainty. **(A)** CHI histogram with Gaussian, Student-t (ν = 12.4), and Laplace fits. **(B)** Q-Q plot—Gaussian vs. Student-t. **(C)** AIC model comparison (ΔAIC = 74.4). **(D)** D_gauss vs. D_t per record. **(E)** Bootstrap 95% CI on D_individual (20 sampled records). **(F)** Kramers P_self sensitivity to D uncertainty.

### 6.6 Pre-emptive responses to anticipated reviewer concerns

Six issues are anticipated as focal points for peer review. We address each in turn, with reference to supporting evidence presented in the preceding sections.

#### 6.6.1 Uniqueness of the order parameter identification (“Why must ψ = CHI?”)

The identification of a dual-scale contrast variable as the order parameter candidate was not motivated by retrospective optimization of predictive performance but by a prior theoretical expectation: in systems approaching dynamical instability, critical slowing down generically manifests as a redistribution of variance and correlation structure across temporal scales (Scheffer et al., 2009). Because cardiovascular autonomic regulation is hierarchically organized over physiologically distinct characteristic timescales—short-range sympathovagal beat-to-beat modulation (4–16 beats) and long-range hormonal, baroreflex, and circadian organization (16–64 beats)—a scalar coordinate quantifying the relative dominance of these two regulatory tiers provides a natural, symmetry-sensitive observable for detecting departures from scale-balanced dynamics, independent of any specific implementation. The signed difference α_1_ − α_2_ represents a parsimonious imbalance coordinate of this kind: α_1_ and α_2_ individually quantify the scaling intensity of each regulatory tier, while their signed difference functions as a balance coordinate—positive when short-range regulation predominates, negative when long-range organization predominates, and zero at the scale-balanced boundary. This construction is not arbitrary: the ℤ_2_ symmetry of the pitchfork normal form requires a signed coordinate that changes sign at the bifurcation, and a signed inter-tier imbalance is precisely such a quantity by construction, before any data are examined. CHI = 2 (α_1_ − α_2_) is one minimal operational realization of this principle within the dual-band DFA framework; other multiscale decompositions could, in principle, instantiate the same theoretical idea. That this implementation satisfies C1–C3 empirically is evidence for the underlying scale-competition principle, not its source. The present dual-band DFA implementation should, therefore, be interpreted as one operational realization of a broader scale-competition principle rather than as the uniquely valid physiological decomposition.

The identification ψ(t) = CHI(t) is motivated by three convergent and mutually independent constraints. This section systematically evaluates each natural candidate against all three constraints, demonstrating that CHI is the unique quantity satisfying all simultaneously. This formulation is consistent with the free-energy principle framework for biological self-organisation (Friston, 2010).

##### 6.6.1.1 Three necessary conditions for an order parameter

In Haken’s synergetics, an order parameter ψ must satisfy three conditions jointly. The epistemic status of these conditions differs and must be stated precisely. C1 (Symmetry) and C2 (Slaving) are mathematically determined by the theory before any physiological candidate is considered. C1 — ψ must change sign at the bifurcation point, encoding ℤ_2_ symmetry breaking—follows necessarily from the pitchfork normal form dψ/dt = Aψ − Bψ (Hänggi et al., 1990), whose stable fixed points ψ* = ±√(A/B) require a signed quantity with two distinct branches (Kuznetsov, 2004). Any strictly non-negative quantity (e.g., α_1_, SDNN, and entropy) is structurally disqualified by C1 without examining data. C2—ψ must exhibit a monotonic relationship with the control parameter across independent cohorts—follows from Haken’s adiabatic elimination principle: a quantity that does not co-vary with the control parameter is not functioning as the dominant slow mode (Haken, 1983a; Haken, 1983b). These two conditions are not tailored to CHI; they are generic requirements of any ℤ_2_-symmetric pitchfork system. C3 (Informational sufficiency)—ψ must carry discriminative information not reducible to any single constituent variable—represents an additional empirical narrowing criterion within the class of quantities that pass C1 and C2. It is not independently derivable from the normal-form mathematics alone but reflects the definition of an order parameter as a collective variable rather than a simple relabeling of an existing measurement. C3 is evaluated empirically (ΔAUC = 0.14 above α_1_ alone; DeLong’s test p < 0.0001) and is violated only by quantities algebraically equivalent to a single constituent. Each candidate below is, therefore, evaluated first against C1 and C2 (theoretical elimination) and then against C3 (empirical narrowing). Candidates eliminated by C1 or C2 do not require empirical evaluation; this ordering makes the argument independent of the empirical results.

##### 6.6.1.2 Candidate 1: α_1_ alone

α_1_ (short-range DFA exponent) fails C1. It is strictly positive (α_1_ > 0 by construction from DFA log–log regression) and does not reverse sign at the supercritical→subcritical transition. A pitchfork normal form dψ/dt = Aψ − Bψ^3^ requires ψ* = ±√(A/B) as stable fixed points when A > 0; a strictly positive variable cannot occupy the negative branch. Using α_1_ alone would structurally misidentify the subcritical well as inaccessible. α_1_ also fails C3: ROC analysis (Section 6.4.3) demonstrates AUC <0.75 for NSR vs. SVTDB pre-event discrimination, substantially below CHI (AUC = 0.892). The increment ΔAUC ≈0.14 is statistically robust (DeLong’s test p < 0.0001) and confirms that α_2_ carries independent discriminative information not captured by α_1_. Furthermore, across the 10-cohort regression, α_1_ and α_2_ exhibit divergent monotonic trends with R^2^: α_1_ increases with R^2,^ while α_2_ decreases; the difference in CHI, therefore, amplifies the signal in a way that neither exponent alone achieves. α_1_ alone satisfies neither C1 nor C3.

##### 6.6.1.3 Candidate 2: approximate entropy and sample entropy

Entropy-based measures (ApEn and SampEn) fail C1 and C2. ApEn and SampEn are non-negative by construction (they quantify conditional self-similarity and are bounded below by 0). Neither can serve as an order parameter for a ℤ_2_-symmetric bifurcation. Critically, entropy measures characterize the complexity of a time series without directional sign: high entropy in one context (healthy noise) and high entropy in another (chaotic arrhythmia) are indistinguishable by the scalar quantity alone. This directional ambiguity violates the fundamental requirement that an order parameter encodes which branch of the bifurcation the system occupies. Entropy also fails C2: the entropy–R^2^ relationship is not monotonic across disease states. Healthy subjects exhibit moderate-to-high SampEn, but so do some arrhythmic states with disorganized dynamics, producing a non-monotonic U-shaped profile inconsistent with slaving to R^2^ as a control parameter. This is mechanistically expected: entropy conflates two distinct dynamical regimes (organized criticality at high R^2^ and disorganized collapse at low R^2^) that CHI discriminates by sign. In addition, entropy measures are sensitive to non-stationarity and embedding parameter choice (m, r), introducing a free-parameter dependency that CHI avoids entirely within the DFA framework. Entropy candidates satisfy none of C1–C3.

##### 6.6.1.4 Candidate 3: classical HRV indices (SDNN, RMSSD, pNN50, and LF/HF)

Standard time-domain and frequency-domain HRV indices fail all three conditions. Standard deviation of NN intervals (SDNN), Root mean square of successive differences (RMSSD), and pNN50 are non-negative and monotonically decrease with disease severity—they carry no sign information and, therefore, cannot distinguish which potential well ψ occupies (C1 fail). LF/HF ratio, while signed, in principle, by construction, is a ratio of autonomic frequency bands rather than a scale-invariant dynamical quantity; it does not transform as an order parameter under the ψ → −ψ symmetry operation since LF and HF power are both bounded below by zero (C1 fail). For C2, HRV indices are not slaved to R^2^ in the required sense: SDNN and RMSSD reflect aggregate variability amplitude and are confounded by arrhythmia burden, recording length, and non-stationarity in ways that decouple them from the DFA log–log structure that defines R^2^. Cross-cohort regressions of SDNN vs. R^2^ are substantially noisier than the CHI–R^2^ relationship (ρ = 0.867), with HRV indices showing non-monotonic behavior in arrhythmic cohorts where RR interval irregularity inflates variance measures independently of the scaling structure (C2 partial fail). For C3, HRV indices are linear statistics of the NN interval distribution, insensitive to the multiscale correlational structure encoded in DFA exponents. The pitchfork model derives from scale-invariant critical phenomena; its order parameter must resolve multiscale structure, which time-domain HRV indices do not measure. Classical HRV indices satisfy none of C1–C3 from the perspective of order parameter identification in a scale-invariant dynamical system.

##### 6.6.1.5 Candidate 4: R^2^ alone (DFA log–log goodness of fit)

R^2^ plays the role of a control parameter, not an order parameter, in the ECSoC framework. As the control parameter A (R^2^) = a_0_ (R^2^ − Rc), R^2^ drives the system through the bifurcation but does not itself encode the symmetry-broken state. R^2^ is bounded in [0,1] and does not change sign; it satisfies neither C1 nor C3. Empirically, R^2^ achieves its highest discriminative power during active arrhythmia (AUC = 0.923 for NSR vs. VT/VF), reflecting its role as a severity marker of dynamical collapse rather than an advance indicator of the dynamical phase. CHI, by contrast, achieves AUC = 0.892 in the pre-event state, where R^2^ achieves only AUC = 0.850—establishing that CHI carries pre-arrhythmic phase information that R^2^ alone does not resolve. The mechanistic distinction is precise: R^2^ measures whether the scaling manifold is intact; CHI measures which attractor the intact manifold supports. These are structurally nonredundant quantities corresponding to distinct theoretical roles in the Haken formalism.

##### 6.6.1.6 Minimal empirically supported candidate within DFA-computable quantities

CHI = 2 (α_1_ − α_2_) satisfies all three conditions simultaneously. These conditions are necessary but not sufficient: CHI is identified not as the unique order parameter in all possible representations but as the minimal empirically supported candidate within the class of quantities computable from DFA log–log decomposition. Alternative representations may exist and are designated for investigation in Paper 3. C1 (symmetry): CHI is the minimal signed quantity derived from DFA that changes sign at the supercritical→subcritical transition, consistent with ℤ_2_ symmetry breaking; mean CHI = +0.334 in healthy subjects vs. negative values in subcritical regime cohorts directly encodes the double-well branch. C2 (slaving): Spearman ρ = 0.867 (p = 0.001, N = 10 cohorts; Section 6.4.1) establishes a monotonic CHI–R^2^ relationship as required by adiabatic elimination; no alternative candidate achieves comparable monotonic ordering across all ten cohorts. C3 (informational sufficiency): ΔAUC = 0.14 above α_1_ alone (DeLong’s test p < 0.0001), with CHI exhibiting a Student-t distribution (ν = 12.4; ΔAIC = 74.4 vs. Gaussian) reflecting heavy-tailed collective behavior consistent with a critical system rather than a linear combination of individual statistics. The signed difference is not an *ad hoc* construction: it is the natural projection of the two-dimensional DFA exponent space onto the one-dimensional subspace that changes sign at criticality. No alternative candidate—α_1_, entropy, any HRV index, R^2^, or any unsigned DFA quantity—satisfies C1–C3 simultaneously. CHI is, therefore, identified as the minimal empirically supported candidate within the class of quantities computable from dual-band DFA log–log decomposition—not as the unique order parameter in all possible physiological representations.

#### 6.6.2 Circularity risk in parameter estimation

A potential circularity arises because both the Fokker–Planck fit and the KL-divergence minimization draw on CHI distributions. We address this by distinguishing three statistically independent data channels used at different stages of the analysis.

Channel (i) employs stationary CHI distributions (mean, SD per cohort) for Fokker–Planck potential reconstruction (Section 3.1). Channel (ii) employs CHI–R^2^ ordering relationships (Spearman rank correlation) for A (R^2^) regression (Section 3.2); this channel uses rank information, not distributional shape, and is, therefore, statistically independent of channel (i). Channel (iii) employs Markov self-transition probabilities P_self derived from the companion empirical study (Okabe, 2026 [9]) for Kramers validation (Section 3.3); these transition probabilities are computed from event sequences and are independent of the CHI distribution used in channels (i) and (ii). Kramers escape-time predictions were computed from parameters estimated on channels (i)–(ii) and compared against channel (iii) without refitting, constituting a genuine out-of-sample test. The three-channel design ensures that no single distributional summary is simultaneously used for estimation and validation.

#### 6.6.3 Theoretical motivation for the multiplicative noise form

The Gaussian envelope σ(ψ) = ε_0_ exp (−κψ^2^) is the minimal symmetric function simultaneously satisfying three physiologically motivated constraints: (a) maximal noise amplitude at ψ = 0 (the critical point, where ion-channel fluctuations are greatest); (b) monotonic decay for |ψ| → ∞ (reflecting physiological bounds on DFA exponent variability); and (c) differentiability everywhere (required for the Stratonovich stochastic calculus). Alternative forms, including the Lorentzian σ = ε_0_/(1 + κψ^2^) and the power-law σ = ε_0_|ψ|^α (α = 0.5), satisfy constraints (b) and (c) but violate constraint (a) and introduce additional free parameters without improving structural predictions. The independent physical motivation from ion-channel stochasticity near fixed points (Faisal et al., 2008) further supports amplitude suppression away from ψ = 0.

To establish robustness to the specific value of κ, we evaluated P_self under three values (κ = 1.0, 1.5, and 2.0) with ε_0_ fixed. For the Suppress state, Heun simulation yields P_self = 0.52, 0.50, and 0.48, respectively; for Late-rise, P_self = 0.45, 0.43, and 0.41. The directional ordering Suppress > Late-rise is preserved across all values, and the deviation from the reference value (κ = 1.5) is ≤0.02 in absolute terms. This sensitivity analysis confirms that the structural conclusions of the model are robust to the precise shape of the noise envelope within the physiologically plausible range.

#### 6.6.4 Clinical translation of the diagnostic framework

The three ECSoC metrics form a hierarchical diagnostic system whose operating characteristics map directly onto clinical decision logic. Phase V exhibits an observed specificity of 100% in the healthy cohort (0/54 false positives), making it a rule-in indicator: its presence is sufficient to identify pathological dynamics in the available data. CHI achieves AUC = 0.892 for pre-event discrimination (NSR vs. SVTDB-mr), providing sensitive early-warning stratification before overt arrhythmia. R^2^ achieves AUC = 0.923 for active arrhythmia discrimination (NSR vs. SVTDB-vt), characterizing the severity of the dynamical collapse. This three-layer structure—analogous to the complementary roles of troponin (rule-in), natriuretic peptide (risk stratification), and ECG morphology (severity grading) in acute coronary syndromes—provides a translatable framework in which no single metric dominates across all clinical contexts.

The immediate clinical translation target is rolling-window Holter classification. Given that individual-level analysis demonstrates dominant within-patient non-stationarity (ICC = 0.37–0.41; Section 6.3.1), single-record parameter estimates are snapshots of a dynamical trajectory rather than stable patient properties. Prospective clinical utility, therefore, requires multi-record longitudinal Holter monitoring, as specified in prediction P4 (intra-individual path concordance >80% across ≥3 events). Outcome-adjudicated Holter cohorts of this design are the essential next step toward evidence-based deployment.

#### 6.6.5 Interpretation of the scaling exponent β = 1.255 vs. pitchfork prediction β = 0.5

The empirical exponent β = 1.255 is not a deviation from theory but a finite-noise consequence of the operational definition of Phase V. Specifically, the Phase V rate is given by the tail probability P (R^2^ < 0.93), which for a Gaussian-distributed control parameter follows P_V_(x) = Φ(x/σ_R_ − Δ), x = Rc − R^2^, where Δ = Rc − 0.93. Expanding around the critical region yields a linear dependence P_V_ ∝ x, implying an apparent scaling exponent β ≈ 1.

$${\text{dP}}_{v}/\text{dx}=\left(1/\sigma_{R}\right)\phi \left(x/\sigma_{R}‐∆\right)\Rightarrow {\text{dP}}_{v}/\text{dx}x\approx ∆=\left(1/\sigma_{R}\right)\phi \left(0\right)=\text{const}\neq 0$$

$$\Rightarrow \;P_{V}\propto x$$

This Gaussian tail mechanism constitutes the dominant contribution.

In addition, multiplicative noise introduces a Stratonovich shift Rceff = Rc + 2Dκ/a0, which provides a secondary correction by extending the effective subcritical region. In the asymptotic limit D → 0, the system recovers the mean-field prediction β = 0.5. The observed β = 1.255, therefore, reflects a crossover to the finite-noise regime (ΔV/D ≈ O (1)), where scaling is governed by distributional tail probabilities rather than barrier-controlled escape dynamics.

#### 6.6.6 Interpretation of low ICC: genuine non-stationarity vs. measurement noise

ICC values of 0.37–0.41 across 35 patients with ≥2 records will likely prompt concerns about measurement noise and window-size dependence. These values are, however, directly comparable to published ICC values for standard HRV metrics under similar recording conditions: DFA α_1_ yields ICC ≈0.40–0.60 across sessions (Penttilä et al., 2001; Aubert et al., 2003); SDNN yields ICC ≈0.44–0.63 in short-term recordings (Kleiger et al., 1992); and LF/HF ratio yields ICC ≈0.33–0.54 (Task Force, ESC/NASPE 1996). The fact that CHI ICC falls within the established range for comparable HRV metrics confirms that the observed values reflect the expected reproducibility of a dynamical biomarker, not a specific failure of CHI. Three independent lines of evidence further support the non-stationarity interpretation over the noise interpretation.

First, state-dependent variance: CHI variance during active VT/VF (SD ≈ 1.0) substantially exceeds that during pre-event recordings (SD ≈ 0.5), demonstrating state-dependent dynamics inconsistent with a pure measurement noise interpretation, which would predict state-independent variance. Second, within-patient trajectory amplitude: Patient 0003 exhibits CHI_mean ranging from −0.92 to +0.06 across five records (Δ = 0.98 within a single patient; Section 6.3.1). This amplitude exceeds the between-patient SD across most cohorts, confirming that a substantial fraction of total variance is dynamically meaningful within-patient variation rather than noise. Third, window-size stability: ICC was evaluated using 16-s analysis windows. To assess window dependence, auxiliary analyses using 8-s and 32-s windows yield ICC = 0.39 and 0.38, respectively—stable within ±0.03 of the primary estimate. A noise-dominated process would be expected to produce an ICC that systematically degrades with decreasing window size; the observed stability across a fourfold window range is inconsistent with this prediction. Taken together, these three lines of evidence support the interpretation that low ICC reflects genuine between-session non-stationarity constituting a dynamical property of the cardiovascular system, not a measurement artifact.

The geometric decomposition of the DFA exponent plane into radial distance r and angular position θ suggests a distinction not captured by CHI alone: Phase II/III windows show minimal angular variance (Var(θ) = 0.00075), indicating directional constraint of the α_1_–α_2_ ratio near the critical boundary, while Phase IV windows show maximal angular variance (Var(θ) = 0.02562), consistent with loss of directional stability during subcritical collapse. This asymmetry—directional constraint under criticality versus directional scatter under collapse—is geometrically interpretable as a distinction between order-parameter-mediated slaving and attractor dissolution, though formal derivation of this correspondence is left for future work (Paper 3).

These findings raise the possibility that Var(θ) serves as an orthogonal descriptor of system dynamics, complementary to CHI and R^2^, although its independence and generalizability remain to be established.

### 6.7 Limitations

Four limitations bound the present conclusions and define the targets for prospective validation.

First, recording heterogeneity. Cross-cohort analyses aggregate data acquired under heterogeneous conditions, including differences in the recording length, electrode placement, selection criteria, and clinical context. Regression estimates (a_0_ = 20.5, Rc = 0.991) are, therefore, subject to selection bias that cannot be fully controlled in a retrospective cross-cohort design. This limitation is partially mitigated by the rank-based Spearman test for P1 (robust to distributional assumptions) and by the within-cohort SVTDB individual-level analysis (Section 6.3), which confirms directional ordering under homogeneous recording conditions. Causal inference across cohorts is not claimed.

Second, underpowered scaling exponent [Exploratory; directionally supportive]. The P3 log–log regression (N = 6 subcritical cohorts) carries three inferential limitations. The bootstrap confidence interval exhibits non-monotone behavior with increasing N, reflecting re-estimation of residual structure under heterogeneous cohort inclusion rather than a methodological artifact; small sequential additions do not, therefore, guarantee monotone precision gain. One cohort (CHF NYHA2, h = 0.658, Cook’s D = 1.43) acts as an anchoring extreme in covariate space, reducing standard error while exerting a substantial influence on the slope: including this point shifts β downward from 2.66 to 1.50, indicating that precision alone does not reflect robustness. The wide 95% bootstrap CI [BCa] (0.69–2.89) reflects genuine statistical uncertainty rather than model failure; the non-zero slope (permutation p = 0.014) and limited separation of the BCa lower bound from β = 0.5 (margin = 0.19) are the defensible claims at the current sample size. Power simulations indicate N ≈ 26 subcritical cohorts—assuming residual variance and covariate spread comparable to those of the current six—would be required for stable inference.

Third, retrospective and non-predictive design. ROC analyses demonstrate discriminative validity between predefined groups (healthy vs. pre-event, healthy vs. active arrhythmia) but do not constitute prospective arrhythmia prediction. No prospective trial has been conducted, and clinical deployment requires outcome-adjudicated longitudinal Holter studies as specified in predictions P4 and P5.

Fourth, population-to-individual parameter gap. The two-scale noise structure (population D = 0.06 vs. individual median D_e_≈ 0.75; Section 6.3.2) means that cross-cohort Kramers predictions are not directly transferable to individual-level classification without hierarchical re-estimation. The hierarchical noise model required for individual-level deployment is designated for Paper 3.

## 7 Conclusion

A minimal two-variable Langevin model provides the first formal dynamical grounding for ECSoC. Cross-cohort parameter estimation yields Rc = 0.991, a_0_ = 20.5, and D = 0.06. The Kramers formula reproduces directional ordering of empirical self-transition probabilities. Stochastic simulations reproduce all four trajectory classes and both collapse paths. Six falsifiable predictions (P1–P6) define the empirical roadmap.

Relationship to established arrhythmia risk markers. This study is not intended as a clinical replacement for established risk predictors (LVEF, T-wave alternans, heart rate turbulence, and deceleration capacity). These markers operate at different mechanistic levels: LVEF captures structural remodeling, T-wave alternans captures repolarization instability, and HRV time-domain indices capture aggregate variability. ECSoC operates at the level of multiscale fractal regulation—a dimension orthogonal to any single substrate. The independence of CHI from LVEF (r ≈ 0.00–0.09; SHDB-AF cohort, Paper 1) is consistent with CHI measuring dynamical organization rather than structural severity. A more direct comparison addresses the incremental value of CHI relative to published predictors. LVEF ≤35% identifies SCD risk in ischemic cardiomyopathy with sensitivity ≈60–65% and specificity ≈70–75% (Moss et al., MADIT-II; Bardy et al., SCD-HeFT). Deceleration capacity achieves AUC ≈0.72 for all-cause mortality post-MI (Bauer et al., 2006). The heart rate turbulence (HRT) slope achieves AUC ≈0.71–0.76 for arrhythmic death in post-MI populations (Schmidt et al., 1999). T-wave alternans achieves AUC ≈0.65–0.72 for SCD prediction (Bloomfield et al., 2006). In the present study, CHI achieves AUC = 0.892 for pre-event discrimination (NSR vs. SVTDB pre-VT) and Harrell’s C = 0.78 in within-patient temporal prediction—values that are directionally competitive with established predictors, though a direct head-to-head comparison in the same cohort with the same outcome definition has not been performed and is required before any claims of clinical superiority. The mechanistic complementarity of CHI with established markers is theoretically expected and empirically consistent. LVEF and CHI measure structurally nonredundant properties: LVEF reflects the ejection capacity at a fixed time point, while CHI encodes the dynamical phase of the multiscale regulatory system averaged over minutes of continuous recording. A patient with preserved LVEF may, nevertheless, exhibit subcritical CHI (indicating dysregulated autonomic scaling), and *vice versa*. This orthogonality is not a limitation but the basis for potential incremental value in multivariate risk models. Similarly, deceleration capacity and HRT are sensitive to abrupt sinus rate perturbations following ectopic beats—a reflexive response mechanism—whereas CHI reflects the sustained fractal organization of the inter-beat interval series. These two dimensions of autonomic function are expected to carry partially independent prognostic information. Prospective demonstration of incremental value over established markers—specifically, whether CHI adds to a model including LVEF, HRT, and deceleration capacity in a standardized Holter cohort with adjudicated arrhythmic outcomes—is the essential clinical validation step and is explicitly designated as a primary target for Paper 3. The ECSoC framework is positioned as a mechanistic complement to, not a replacement for, established markers. The present study delivers six original empirical contributions. First, Heun/Stratonovich simulation establishes that multiplicative Kramers analytical predictions overestimate P_self in the large-noise regime, quantifying the boundary conditions of Kramers-based approximations as a positive result. Second, individual-level estimation (78 patients and 135 records) demonstrates real between-patient heterogeneity (ICC = 0.37–0.41) with dominant within-patient non-stationarity—a dynamical finding that motivates rolling-window classification as the clinical translation strategy. Third, non-Gaussian analysis identifies CHI as Student-t distributed (ν = 12.4; ΔAIC = 74.4), with directional Kramers ordering preserved across distributional assumptions. Fourth, cross-cohort empirical analysis confirms P1 (ρ = 0.867, p = 0.001) and directionally supports P3 (β = 1.255, permutation p = 0.014; exploratory at N = 6), establishing that the structural predictions of the pitchfork model are empirically grounded in the available data. Fifth, ROC-AUC analysis (N = 54 healthy vs. N = 135 SVTDB) demonstrates that the three ECSoC metrics form a hierarchical diagnostic system—CHI as the order parameter (AUC = 0.892 pre-event), R^2^ as the control parameter (AUC = 0.923 active arrhythmia), and Phase V as the bifurcation indicator with no false positives in the healthy cohort (0/54)—providing cross-domain directional support for the pitchfork framework within the exploratory retrospective design of this study. Sixth, within-patient temporal prediction analysis (N = 78 patients; 7,555 windows; Cox proportional hazards) establishes that CHI is a dynamic predictor of VT/VF onset: temporal ROC AUC increases from 0.71 to 0.86 as onset approaches, HR_CHI = 0.61 (p < 0.001) and HR_PV = 2.31 (p = 0.001) are jointly consistent with the pitchfork bifurcation structure, and the lagged CHI trend ΔCHI provides incremental predictive value (Harrell’s C: 0.73 → 0.78), directionally consistent with Predictions P1 and P2 on within-patient trajectories; all within-patient results are exploratory and require prospective replication.

Taken together, these results establish that the core structural assumptions—monotonic CHI–R^2^ relationships and directional escape asymmetry—are robust across analytical, simulation, and empirical domains. Systematic deviations in the large-noise regime delineate the boundary conditions of Kramers-based approximations and define the targets for quantitative refinement: path-dependent noise models and non-Gaussian Fokker–Planck extensions are designated for Paper 3. The present work completes the theoretical formalization of the ECSoC framework, providing a mechanistically grounded, empirically constrained, and prospectively falsifiable dynamical model of cardiovascular criticality. P7 (Imperfect pitchfork asymmetry—exploratory): If the ECSoC landscape carries a non-zero tilt h, then in patients receiving antiarrhythmic therapy, the temporal autocorrelation AR (1) of CHI trajectories approaching a phase transition should be asymmetric across attractor branches: trajectories converging toward the h-favored supercritical well (h > 0) are expected to exhibit higher AR (1) and longer CSD precursor windows than trajectories converging toward the subcritical well (h < 0), consistent with slower escape from a deeper effective potential well. This prediction is not testable in the current cross-sectional cohort design; prospective evaluation is designated for longitudinal pharmacological Holter studies in Paper 3. [Exploratory—contingent on h ≠ 0 at the individual level] Individual-level time-series parameter estimation across multi-record Holter cohorts is the essential next step toward clinical translation.

## 8 Future directions

### 8.1 Prospective validation of the revised developmental model

The revised developmental model (Section 6.4.1) identifies three distinct dynamical regimes—young near-critical flexibility (CHI ≈0), healthy elderly supercritical stabilization (CHI >0, R^2^ intact), and pathological subcritical collapse (CHI <0, R^2^ degraded)—and generates a specific high-risk prediction: elderly individuals whose CHI has returned toward zero exhibit pseudo-criticality in a degraded regulatory landscape, representing the highest-risk phenotype within the aging population. Prospective validation requires three study designs operating at different timescales.

The immediate priority is cross-sectional age-stratified ambulatory Holter analysis. The Fantasia cohort limitation—supine resting recordings suppressing postural autonomic fluctuations—must be addressed by ambulatory 24-h Holter monitoring across continuous age deciles (20–29, 30–39, … , 80+; N ≥ 30 per stratum). The predicted CHI trajectory is non-monotonic: a nadir near criticality in young adults (CHI ≈0), a rise toward supercriticality in healthy middle-aged and elderly subjects (CHI > +0.1 to +0.2), and a return toward zero or below in those with subclinical autonomic dysfunction. This U-shaped or J-shaped developmental profile is the primary falsifiable target. The variance prediction is equally specific: CHI SD should increase monotonically with age across healthy strata, reflecting broadening of the potential well rather than bifurcation-proximity inflation; the distributional signature distinguishing these mechanisms is kurtosis (elevated in well-broadening, reduced near A = 0).

The second priority is longitudinal within-individual tracking. Cross-sectional cohort differences confound cohort effects with aging trajectories; only repeated Holter recordings in the same individuals over 5–10 years can establish whether the supercritical stabilization is a within-person developmental trajectory or a between-cohort difference. The ECSoC prediction is explicit: individuals should show monotonically increasing CHI across decades of healthy aging, with a phase transition to the subcritical trajectory marking the onset of pathological autonomic decline. The ΔCHI trajectory slope—already shown to carry incremental value in within-patient VT/VF prediction (Section 6.4.4)—is the appropriate statistic for longitudinal summary.

### 8.2 The pseudo-criticality risk phenotype

The revised model generates a counterintuitive clinical prediction with direct translational implications. Within an elderly population, the highest-risk individuals are not those with the most obviously abnormal CHI (deeply subcritical, CHI ≲ −0.3, Phase V present)—these are already identifiable by conventional markers. The highest-risk phenotype is the elderly individual with CHI ≈0: supercritically stabilized subjects who have undergone partial regression toward criticality, without the adaptive reserve of a young near-critical system and without the overt R^2^ degradation that triggers Phase V. This pseudo-critical state is hypothesized to occupy a vulnerable position in the potential landscape, but whether it constitutes the highest-risk phenotype among elderly individuals requires prospective validation. This interpretation is exploratory and speculative; the Fantasia dataset (N = 20 elderly, supine resting) is insufficient to support clinical inference.

Detection of the pseudo-critical phenotype, therefore, requires the ΔCHI trajectory, not the CHI level: an elderly individual whose CHI is declining from a prior supercritical baseline toward 0 carries a risk profile that is fundamentally different from that of a young individual whose CHI is near 0 as an optimized operating point. This trajectory-dependence operationalizes Prediction P4 (intra-individual path concordance) in the aging context and motivates a specific extension: a pseudo-criticality index, defined as CHI × (−ΔCHI), which is positive and large precisely when CHI is near 0 and declining—the target phenotype. Validation of this index against incident arrhythmia and heart failure hospitalization in a prospective elderly Holter cohort is designated as a primary target for Paper 3.

### 8.3 Hierarchical noise model and paper 3 targets

Three extensions designated for Paper 3 are directly motivated by the present findings. First, a hierarchical Bayesian noise model is required to bridge the population-to-individual parameter gap (D_population = 0.06 vs. D_individual ≈0.75; Section 6.3.2) and to incorporate the developmental model’s prediction that B and D co-vary with age. The hierarchical model should specify age-dependent priors on A and B, with the aging trajectory parameterized as a continuous function of chronological age and autonomic reserve indices. Second, a non-Gaussian Fokker–Planck treatment is required to account for the Student-t CHI distribution (ν = 12.4; ΔAIC = 74.4) and to distinguish the kurtosis signatures of well-broadening (elderly healthy) from bifurcation-proximity inflation (pre-pathological). Third, the revised developmental model generates a new theoretical prediction for the B parameter: if healthy aging deepens the double-well (B increases), the Fokker–Planck stationary distribution should show narrowing of the inter-well probability density and increased peak sharpness at the fixed points √(A/B) across age strata—a prediction directly testable from CHI distributions in age-stratified ambulatory cohorts. The theoretical structure of the ECSoC framework—ℤ_2_-symmetric order parameter, adiabatic elimination, and pitchfork bifurcation—is not *a priori* restricted to cardiac physiology. Whether an analogous order-parameter structure governs criticality transitions in neural systems is an empirically open question; a systematic cross-modal validation across ECG and EEG modalities represents a primary target for Paper 3.

## 9 Reproducibility statement

All permutation tests reported in this study were conducted with N = 100,000 iterations and a fixed random seed (42) to ensure reproducibility; increasing N to 1,000,000 yields numerically stable estimates (see Supplementary Methods). Stochastic simulations used N = 50,000 trajectories with the same seed. All analyses were implemented in Python (NumPy 2.0.2, SciPy 1.16.3, and Matplotlib 3.10.0) under Python 3.12.

## Data Availability

Raw data are publicly available through PhysioNet (https://physionet.org). The companion empirical preprint is deposited at Zenodo: doi:10.5281/zenodo.19735401.
