# Supplementary material for: Empirically constrained order parameter dynamics in cardiovascular criticality: a synergetic Langevin framework for arrhythmic transitions with cross-cohort parameter estimation and Kramers escape-time validation
Source: Front Netw Physiol. 2026 Jul 9;6:1865256. doi: 10.3389/fnetp.2026.1865256 (PMC13391250; doi:10.3389/fnetp.2026.1865256)
Supplement: Supplementary file 1 [file Table1.docx]

# **Statistical Supplement: Benchmark Validation and Autocorrelation Falsification**

*Manuscript: Empirically Constrained Order Parameter Dynamics in Cardiovascular Criticality*

Dataset: SVTDB (PhysioNet) — 78 patients, 135 MR records, 135 VT/VF records

Analysis: Bootstrap N = 2,000; seed = 42; 5-fold stratified CV

# **① AUC / C-index Significance Tests**

**Methods.** All AUC values derive from 5-fold stratified cross-validation (CV). Significance of pairwise AUC differences was assessed by percentile bootstrap (N = 2,000 resamples, seed = 42; DeLong's method cannot be applied to CV predictions). Two baselines were pre-specified: (1) AR(1)-analogue — lagged CHI from the first half of each MR record; (2) RR-autocorrelation — the lag-1 Pearson autocorrelation of the raw RR time series, representing naïve autoregressive signal.

| **Model** | **CV-AUC** | **vs AR(1) ΔAUC** | **95% Bootstrap CI** | **p-value** | **Interpretation** |
| --- | --- | --- | --- | --- | --- |
| AR(1) baseline (lagged CHI) | 0.522 | — | — | — | Reference |
| CHI alone | 0.636 | +0.114 | [+0.052, +0.173] | 0.0005 | Significant |
| R² alone | 0.767 | +0.245 | [+0.146, +0.343] | < 0.001 | Highly significant |
| **ARX (CHI+R²+PV)** | **0.827** | **+0.304** | **[+0.229, +0.377]** | **< 0.001** | **Highly significant** |
| RR-autocorr baseline | 0.597 | — | — | — | Reference (AC) |
| **ARX vs RR-autocorr** | **0.827** | **+0.230** | **[+0.130, +0.322]** | **< 0.001** | **Beyond autocorr.** |

**Interpretation.** The ARX model (CHI + R² + Phase V) achieves CV-AUC = 0.827, significantly exceeding both the AR(1) baseline (ΔAUC = +0.304; p < 0.001) and the RR-autocorrelation baseline (ΔAUC = +0.230; p < 0.001). CHI alone also significantly outperforms the AR(1) baseline (ΔAUC = +0.114; p = 0.0005), confirming that the DFA-based order parameter captures predictive information beyond simple autoregressive structure.

# **② Explicit Falsification of the Autocorrelation Hypothesis**

The central alternative explanation for the observed predictive performance is that CHI and R² merely capture temporal autocorrelation in the RR series — i.e., that the ECSoC framework provides no information beyond what is already encoded in the memory structure of the interbeat interval sequence. Four independent tests jointly falsify this hypothesis.

| **Test** | **Metric** | **Statistic** | **p-value** | **Conclusion** |
| --- | --- | --- | --- | --- |
| CHI vs RR-autocorr correlation | Spearman ρ | 0.049 | 0.423 | CHI ≠ autocorr surrogate |
| Partial r: CHI–VT (controlling RR-AC) | Pearson r | 0.270 | < 0.001 | CHI explains VT beyond AC |
| Partial r: CHI–VT (controlling lag-CHI) | Pearson r | 0.281 | < 0.001 | CHI ≠ AR(1) in content |
| ΔCHI (VT−MR) within-patient | Paired t-test | t = 4.04 | 0.0001 | Arrhythmia-specific change |
| ΔR² (VT−MR) within-patient | Paired t | t = −7.82 | < 0.001 | Arrhythmia-specific change |
| CHI AUC vs lagged CHI AUC | Bootstrap | ΔAUC = +0.101 | 0.001 | Current CHI ≠ lag-CHI |

**Interpretation.** The Spearman correlation between CHI and RR lag-1 autocorrelation is near zero (ρ = 0.049, p = 0.423), demonstrating that these measures are statistically orthogonal — CHI is not a surrogate for autocorrelation. The partial correlations of CHI with VT status remain highly significant after controlling for both RR-autocorrelation (r = 0.270, p < 0.001) and lagged CHI itself (r = 0.281, p < 0.001). Within-patient paired comparisons show that ΔCHI (t = 4.04, p = 0.0001) and ΔR² (t = −7.82, p < 0.001) are both significant. Together, these results confirm that the ECSoC order-parameter trajectory captures arrhythmia-specific dynamical information that cannot be attributed to autocorrelation, marginal CHI distribution, or ordinary autoregressive structure.

# **③ Shuffle Control Experiment**

**Methods.** Two shuffle controls were implemented to verify that the observed signal depends on temporal structure rather than marginal distributions. (1) *Within-record temporal shuffle:* the CHI value of the full record was compared to the CHI of the first-half segment; the observed difference (Δ = −0.128) was compared to N = 1,000 permutations in which the first/second assignment was randomly flipped within each patient. (2) *Label shuffle:* patient MR/VT labels were permuted to verify that the within-patient ΔCHI could not arise by chance.

| **Condition** | **Observed statistic** | **Shuffled (N=1,000)** | **Permutation p** |
| --- | --- | --- | --- |
| CHI temporal gradient (full − first-half) | Δ = −0.128 | 0.000 ± 0.041 | < 0.001 → temporal structure required |
| Within-patient ΔCHI (VT − MR) | +0.354 ± 0.769 | 0.000 ± 0.087 (label-shuffle) | 0.0001 → not a random label effect |

**Interpretation.** The temporal CHI gradient (full − first-half = −0.128) has permutation p < 0.001 against the shuffled null distribution (mean = 0.000 ± 0.041). This establishes that the temporal trajectory of CHI — not merely its marginal distribution — contains the predictive signal. A pure autocorrelation artefact, which preserves marginal properties under shuffling, is therefore excluded.

## **Computational Details**

Dataset: SVTDB (PhysioNet, spontaneous-ventricular-tachyarrhythmia-database-1.0). N = 78 patients; 135 MR (monitoring) records and 135 VT/VF records. DFA computed on full records (N ≈ 1,000 beats per record); scales 4–64 beats (log-spaced, 20 points); α₁ fitted over scales 4–16, α₂ over scales 16–max(N/4, 64); CHI = 2(α₁ − α₂); Phase V threshold R² < 0.93. AR(1)-analogue: CHI from first half of each MR record. RR-autocorrelation: Pearson lag-1 autocorrelation of raw RR series. Bootstrap: N = 2,000 resamples, seed = 42. CV: 5-fold stratified, LogisticRegression (C = 1.0, StandardScaler). All analyses: Python 3.12, NumPy, scikit-learn, SciPy.
